# CTG clade-specific proteins of the RSC chromatin-remodeling complex regulate cell cycle progression of a critical priority fungal pathogen, *Candida albicans*

**DOI:** 10.1128/msphere.00084-26

**Published:** 2026-03-30

**Authors:** Ankita Joshi, Gayatri Brahmandam, Harini Kannan, Shilajit Roy, Sandhya Subramanian, Amartya Sanyal, Santanu Kumar Ghosh

**Affiliations:** 1 Department of Biosciences and Bioengineering, Indian Institute of Technology Bombay, Powai, Mumbai, India; 2 Department of Biological Sciences, Birla Institute of Technology and Science, Pilani, Hyderabad Campus, Hyderabad, Telangana, India; CNRS-Inserm-Université Côte d'Azur, Nice, France

**Keywords:** RSC complex, chromatin-remodeling, fungal pathogen, *Candida albicans*, cell cycle, virulence

## Abstract

The remodels the structure of chromatin (RSC) and the homologous chromatin-remodeling complexes are known to regulate cell cycle progression in various organisms, including *Saccharomyces cerevisiae*, *Drosophila*, and *Homo sapiens*. In this work, we characterized the role of two novel CTG clade-specific proteins (Nri1 and Nri2) of the RSC complex in the regulation of cell cycle progression in a critical priority fungal pathogen, *Candida albicans*. We observed that Nri1, alone or along with Nri2, regulates cell cycle progression at multiple stages. The *nri1Δ/Δ* and *nri1Δ/Δ nri2Δ/Δ* mutants exhibited transient cell cycle arrest, defective spindle morphology, and cytokinesis. Transcriptomic analysis supported these mutant phenotypes and indicated a broad role of Nri proteins in the cell cycle. From our results, we conclude that Nri proteins are crucial for *C. albicans* proliferation and fitness.

**IMPORTANCE:**

The composition of the essential RSC chromatin-remodeling complex exhibits species-specific divergence, harboring unique subunits with distinct functions. In this study, we report that two fungal CTG clade-specific proteins of the *C. albicans* RSC complex, namely Nri1 and Nri2, can promote *C. albicans* fitness by regulating its cell cycle progression at multiple stages. Fitness defects, along with stressor sensitivity and differential expression of the genes regulating pathogenesis in the *nri* mutants, indicate the potential of the Nri proteins in the regulation of *C. albicans* virulence.

## INTRODUCTION

*Candida albicans*, categorized as a critical priority fungal pathogen by the World Health Organization, inhabits various niches in the human host as a harmless commensal (1). It is present in the oral cavity, in the gastrointestinal and genitourinary tracts, and on the skin of the majority of the population (2–6). However, in the case of compromised host immunity and disbalanced microbiota due to various reasons, it can cause mucosal infections or life-threatening systemic infection (7–10). *C. albicans* uses a diverse range of strategies to improve its survival in the host niches, ultimately increasing virulence. It is a polymorphic fungus; it can adhere and invade host mucosa, form biofilms, generate “beneficial aneuploidy,” and has the potential to develop resistance against antifungal drugs (11–13). Most of these pathogenic attributes fundamentally rely on transcriptional regulation, where modulation of chromatin on demand plays a crucial role. Consequently, the chromatin factors such as histone-modifying enzymes and ATP-dependent chromatin-remodeling complexes have emerging roles in fungal pathogenesis (14, 15). Histone modifications impart biological functions through *cis* effects on the chromatin itself, and through *trans* effects, they recruit various “reader proteins,” including chromatin-remodeling complexes, to alter the chromatin accessibility by repositioning, exchanging, or removing the nucleosomes (16, 17).

Various reports highlight the importance of chromatin factors in *C. albicans* biology. Histone acetylase Rtt109 is crucial for nucleosome assembly in the S phase, and *C. albicans* lacking *RTT109* exhibits hypersensitivity to the DNA-damaging agent hydroxyurea with the activation of the DNA damage response (18). *C. albicans* exhibits defective spindle morphology and anaphase progression when histone deacetylases Hst3 activity is inhibited (19). In this organism, Sir2 histone deacetylase is also reported to regulate rDNA stability and mitotic exit (20). Besides regulating cell cycle progression, the histone modifiers are also known to influence cell wall integrity, morphological transitions, genome stability, stress response, and virulence in *C. albicans* (18, 19, 21–24). Exchange of canonical histones with timely deposition of histone variants H3V^CTG^ and H2A.Z was also shown to contribute to morphological transitions and biofilm formation in *C. albicans* (25–27). Similarly, the chromatin-remodeling complexes also regulate several crucial cellular functions in various organisms. SWI/SNF superfamily of remodelers, predominantly the RSC chromatin remodeling complex, controls transcriptional activation and repression (28–30), mitotic progression (31, 32), DNA damage repair (33–35), cytoskeletal organization (36), kinetochore (KT) clustering (32), cohesion (32, 37–39), and chromosome segregation (40) in fungal organisms.

Earlier, through mass spectrometry-based identification of the *C. albicans* RSC chromatin-remodeling complex, we discovered the presence of two novel CTG-clade-specific subunits, namely Nri1 (Novel RSC Interactor 1) and Nri2 (Novel RSC Interactor 2). In that work, we showed that the *nri1Δ/Δ* mutant exhibited growth defects at standard growth conditions, increased susceptibility to various stressors, and a hyphal induction defect. On the other hand, *nri2Δ/Δ* only exhibited thermosensitivity (30). Given the key roles of several core subunits of the RSC complex in *C. albicans* proliferation and pathogenicity (30, 32, 41–43), we hypothesized that CTG clade-specific Nri proteins would also significantly influence *C. albicans* biology and have the potential to be ideal drug targets. In this work, we demonstrate that the *nri* mutants are defective in cell cycle progression. Moreover, they show a KT organization defect, impaired spindle morphology, and altered chromatin compaction. RNA sequencing (RNA-seq) analysis of *nri* mutants revealed differential expression of several genes involved in those processes, supporting the observed phenotypes. Altogether, we conclude that the Nri proteins indeed can regulate a broad range of cellular processes. Rapid adaptation through proficient stress response, increased drug resistance cases, and limited antifungal treatment options due to associated host toxicity (44) advocates the dire need for developing novel therapeutic targets. Chromatin-remodeling proteins, being regulators of fungal fitness by means of diverse pathways, can in fact limit both fungal growth and adaptation, and hence, the virulence. In this context, our work postulates the role of fungal CTG clade-specific RSC complex proteins in regulating *C. albicans* fitness, highlighting their importance as therapeutic targets.

## RESULTS

### *nriΔ/Δ nri*2*Δ/Δ* double mutant is synthetically sick, indicating the genetic interaction between the *NRI* genes

**Fig 1:**
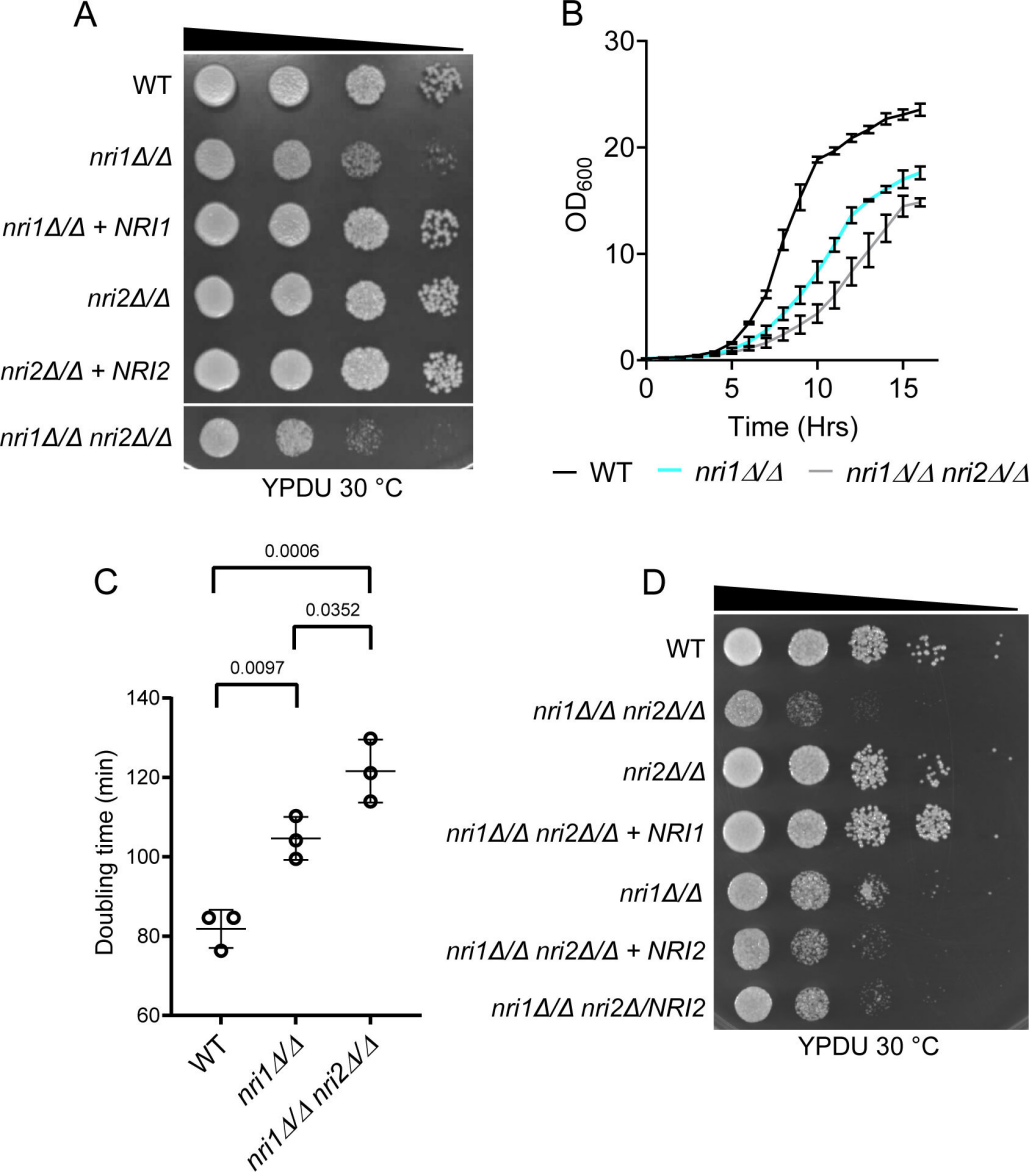
*nri1Δ/Δ nri2Δ/Δ* double mutant shows a synthetic sick phenotype. (**A**) Image showing growth of 10-fold serially diluted cells of the indicated strains spotted on the YPDU plate and incubated at 30°C for 48 h and imaged. (**B**) The growth curve was measured for WT, *nri1*Δ/Δ mutant, and *nri1*Δ/Δ *nri2*Δ/Δ double mutant strains at standard growth conditions. OD_600_ was recorded every 60 min. Mean values with standard deviation from three biological replicates were plotted. (**C**) Doubling time of the strains was calculated from the growth curve. Data from three biological replicates are plotted. Error bars indicate standard deviation. Statistical analysis was performed by one-way ANOVA. (**D**) Image showing growth of 10-fold serially diluted cells of the indicated strains spotted on the YPDU plate and incubated at 30°C for 48 h and imaged. A *P*-value of < 0.05 was considered significant. Only significant *P*-values are mentioned in the graphs.

We reported earlier that the *nri1Δ/Δ* mutant had growth defects under standard growth conditions, and it was hypersensitive to various stress-inducing agents mimicking physiologically relevant stresses; on the contrary, the *nri2Δ/Δ* mutant only displayed thermosensitivity (30). To determine the impact of the combined loss of *NRI1* and *NRI2* genes on *C. albicans*, we constructed a double mutant of these genes (*nri1Δ/Δ nri2Δ/Δ*). Similar results were obtained for *nri1Δ/Δ* and *nri2Δ/Δ* single mutants as reported in a previous study (30) (Fig. 1A; Fig. S1A), whereas the *nri1Δ/Δ nri2Δ/Δ* double mutant exhibited a severe growth defect under standard growth conditions (Fig. 1A and B). The doubling times for *nri1Δ/Δ* and *nri1Δ/Δ nri2Δ/Δ*, calculated based on the growth curve, were 104.6 min and 121.6 min, respectively, which were significantly higher than that of the wild-type (WT) strain that took 81.84 min to double (Fig. 1C). The observed synthetic sick phenotype of the *nri1Δ/Δ nri2Δ/Δ* double mutant confirmed a genetic interaction between the *NRI* genes. As expected, the *nri1Δ/Δ nri2Δ/Δ* double mutant also displayed hypersensitivity to various physiologically relevant stress conditions, similar to the *nri1Δ/Δ* mutant (Fig. S1B). All the mutants exhibited thermosensitivity at 42°C, although only the *nri1Δ/Δ nri2Δ/Δ* double mutant showed sensitivity at 37°C (Fig. S1C). We then constructed phenotype rescue strains of *nri1Δ/Δ nri2Δ/Δ* double mutant by reintroducing a single copy of either *NRI1* or *NRI2* gene. While *NRI1* reintegration completely restored the growth rate and the strains grew similar to *nri2Δ/Δ* mutant, *NRI2* reintegration could not fully rescue the growth defect, probably indicating that the *NRI2* gene is haploinsufficient under *nri1Δ/Δ* background (Fig. 1D). As *nri2Δ/Δ* single mutant displayed no major phenotypes (except thermosensitivity as reported earlier), we then focused on *nri1Δ/Δ* single mutant and *nri1Δ/Δ nri2Δ/Δ* double mutant for further characterization.

### Absence of Nri proteins causes global alterations in the transcriptome profile

**Fig 2:**
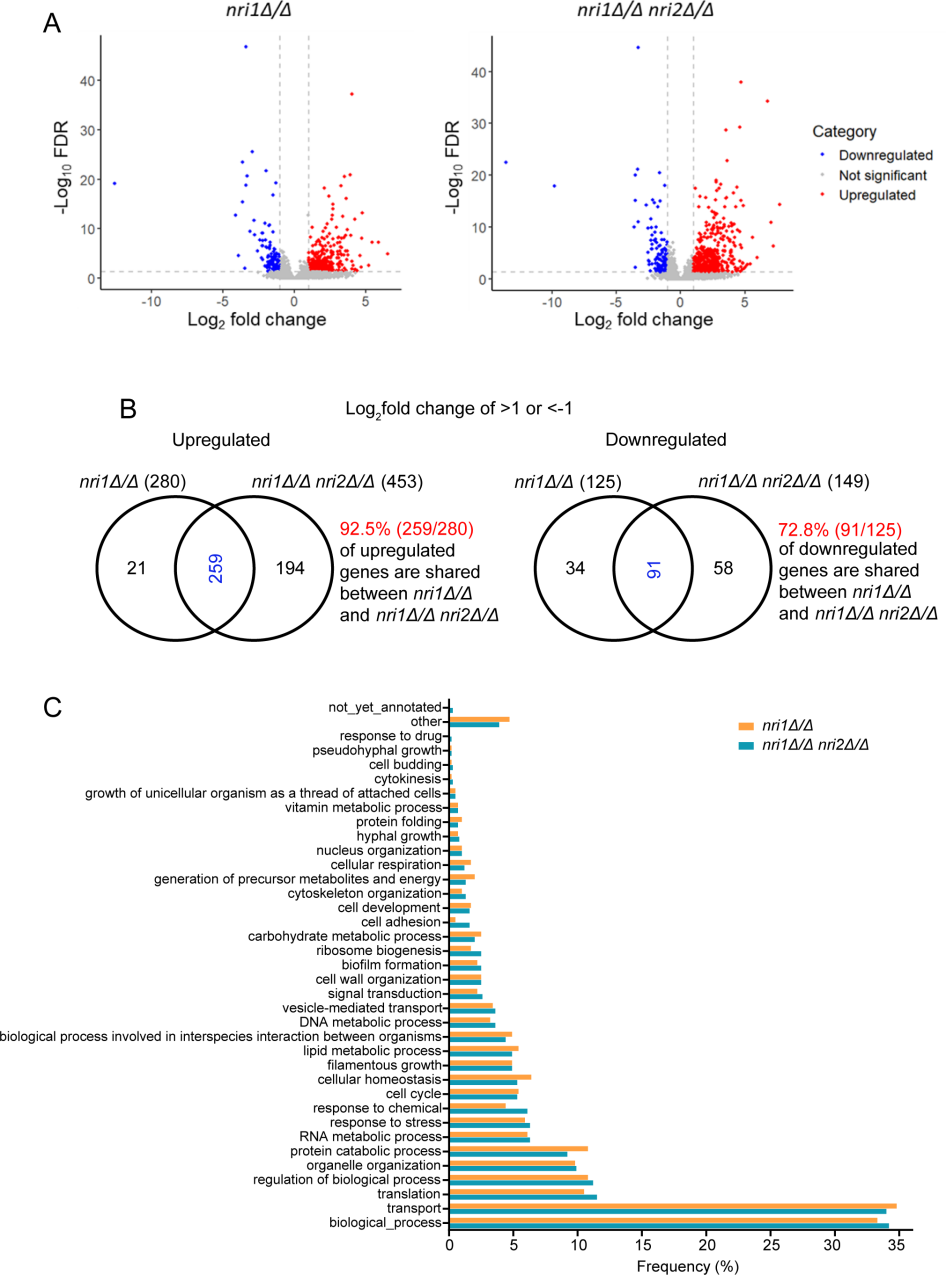
nri mutants exhibit global alterations in the transcriptome profile. (**A**) Volcano plot representations of the differentially expressed genes (DEGs) for *nri1Δ/Δ* w.r.t. WT (*nri1Δ/Δ*) and *nri1Δ/Δ nri2Δ/*Δ w.r.t. WT (*nri1Δ/Δ* nri2Δ/Δ) samples. Significantly altered genes with the threshold of −1<log2(Fold Change)>1 and FDR (adjusted *P*-value) <0.05 are highlighted in blue and red colors, respectively. (**B**) Venn diagram indicating overlap between the upregulated and downregulated genes in *nri1Δ/Δ* and *nri1Δ/Δ nri2Δ/Δ* mutants. Numbers in the parentheses denote the total number of up-regulated and down-regulated DEGs in the indicated mutant strains. (**C**) Gene ontology (GO) analysis of DEGs in *nri1Δ/Δ* and *nri1Δ/Δ nri2Δ/Δ* samples. The Y-axis indicates various GO terms of the biological processes, and the X-axis indicates percentage of the DEGs encompassing that GO term.

**Fig 3:**
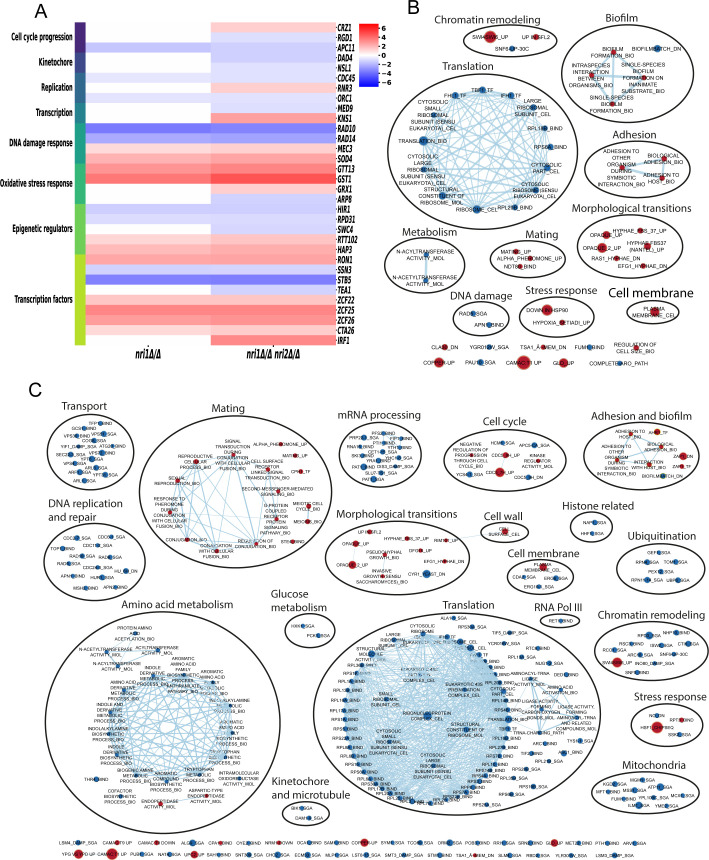
*nri* mutants exhibit misregulation of gene sets corresponding to various biological processes. (**A**) Heat map of DEGs for the indicated samples in various processes relevant to cell proliferation and virulence. Color scale represents log_2_ fold change for the DEGs. Network-based visualization of GSEA results using the EnrichmentMap feature of Cytoscape for (**B**) *nri1Δ/Δ* and (C) *nri1Δ/Δ nri2Δ/Δ* mutants. For both **B and C**, the size of node (gene set) is proportional to the number of DEGs in that node. Nodes with the down-regulated and up-regulated genes are shown by blue- and red-filled circles, respectively. Light blue lines connect different clusters whose thickness is based on the number of overlapping genes. Clusters belonging to a similar functional category are encircled in black.

As Nri proteins are part of the RSC chromatin-remodeling complex that has myriad functions (30, 32), high-throughput RNA-seq analysis was performed to understand the impact of the absence of *NRI1* and *NRI2* genes on the *C. albicans* transcriptome profile (Fig. 2). With the absolute fold change of 2 and false discovery rate (FDR) threshold of <0.05 (adjusted *P*-value), the absence of the *NRI1* gene resulted in 280 up- and 125 down-regulated transcripts, accounting for 6.26% (405/6,468) of the total transcripts in haplotype A of *C. albicans* strain SC5314 assembly 22. The combined loss of *NRI1* and *NRI2* genes had a relatively greater impact on transcriptomic changes with 453 up- and 149 down-regulated transcripts, comprising 9.3% (602/6,468) of the total transcripts, corroborating genetic interaction between the two genes (Fig. 2A and B). Gene ontology (GO) analysis, using GO Slim Mapper tool, indicated that the differentially expressed genes (DEGs) regulated various growth or fitness-related processes such as “transport,” “translation,” “organelle organization,” “protein catabolic process,” “RNA metabolic process,” “cell cycle,” “cellular homeostasis,” “ribosome biogenesis,” and “cytoskeleton organization.” Apart from this, DEGs were also from the processes that influence the virulence of the organism, such as “response to stress,” “filamentous growth,” “interspecies interaction,” “cell wall organization,” “biofilm formation,” and “cell adhesion” (Fig. 2C). This analysis indicates that the Nri proteins regulate both growth and virulence attributes. Based on the growth defect observed in *nri* mutants (Fig. 1), we hypothesized that the expression of the cell cycle progression-related genes might be misregulated in the mutants. In addition, as RSC mutants from other and this organism are known to regulate DNA damage, stress responses, and epigenetic and transcription factors, we shortlisted those DEGs that are related to these pathways (Fig. 3A). Thus, to pinpoint the genes misregulated in the mutants, detailed analysis of the DEGs revealed a significant downregulation of *APC11* transcript, an ortholog of the anaphase-promoting complex component, in both *nri1Δ/Δ* and *nri1Δ/Δ nri2Δ/Δ* mutants compared to WT. DNA replication-regulating genes *RNR3*, *CDC45*, and *ORC1*, transcription regulatory gene *KNS1*, KT protein-encoding genes *NSL1* and *DAD4*, and mediator complex subunit encoding gene *MED9* were also found to be differentially expressed in the *nri1Δ/Δ nri2Δ/Δ* mutant. Out of these DEGs, *CDC45* and *ORC1* also showed significant downregulation in *nri1Δ/Δ* single mutant. It is worth mentioning that downregulation of *CDC45*, *ORC1*, *NSL1*, and *MED9* was observed at a fold change cutoff of 1.5-fold, a commonly used parameter in several studies (30, 45–47). Several oxidative stress-related genes were differentially expressed in both mutants, supporting the observed H_2_O_2_ sensitivity (Fig. S1B). In *S. cerevisiae*, *APC11* is involved in anaphase entry and cell cycle exit, *ORC1* and *CDC45* genes are involved in DNA replication, *RNR3* regulates dNTP synthesis, *KNS1* regulates RNA polymerase III transcription, *NSL1* and *DAD4* are required for chromosome segregation, and *MED9* regulates RNA polymerase II activity. As these genes are highly conserved, they are presumed to do similar functions in *C. albicans*. Thus, differential expression of these genes indicates the involvement of the Nri proteins in the regulation of cell cycle, DNA replication, and transcription processes in *C. albicans*. Apart from this, several transcription factors and epigenetic regulators, such as *RON1*, *ZCF25*, *ZCF26*, and *HIR1*, exhibited differential expression, suggesting the possible indirect regulatory roles of *Nri1* and *Nri2* proteins (Fig. 3A). Further in-depth investigation of the RNA-seq data sets was performed with Gene Set Enrichment Analysis (GSEA). GSEA results indicated that the absence of *NRI1* and *NRI2* caused misregulation of a broad range of processes, and the *nri1Δ/Δ nri2Δ/Δ* double mutant affected more processes compared to the *nri1Δ/Δ* mutant, explaining the synthetic sick phenotype of the double mutant. Strikingly, in contrast to the observation that DEGs in both *nri1Δ/Δ* and *nri1Δ/Δ nri2Δ/Δ* mutants exhibited a higher number of upregulated genes compared to downregulated genes with respect to WT strain, GSEA analysis revealed that the absence of *NRI* genes mostly resulted in downregulation of several processes, except the adhesion, morphological transition, biofilm, and mating clusters that were upregulated. The major cluster that was downregulated in both *nri1Δ/Δ* and *nri1Δ/Δ nri2Δ/Δ* mutants harbors translation- and ribosome-related processes. The *nri1Δ/Δ* mutant also exhibited misregulated clusters including cell membrane, chromatin remodeling, DNA damage, stress response, and metabolism (Fig. 3B). On the other hand, the cell wall cluster was upregulated, while cell membrane, DNA replication and repair, transport, mRNA processing, metabolism, chromatin remodeling, ubiquitination, mitochondria, and KT-microtubule-related clusters were downregulated in the *nri1Δ/Δ nri2Δ/Δ* double mutant (Fig. 3C). Additionally, to understand the functional contribution of the Nri proteins within the CaRSC complex, we compared this GSEA data with what was obtained earlier (30) from *C. albicans* cells lacking Sth1, the main ATPase subunit of the CaRSC complex. Both *nri* mutants and CaSth1-depleted cells exhibited similarities in misregulation of mitochondria, stress response, cell wall, and chromatin remodeling-related processes. Overall, the GSEA data corroborate with some of the tested phenotypes of *nri* mutants and highlight that the *NRI* genes regulate numerous processes related to *C. albicans* fitness.

### *nri* mutants exhibit altered cell cycle progression

**Fig 4:**
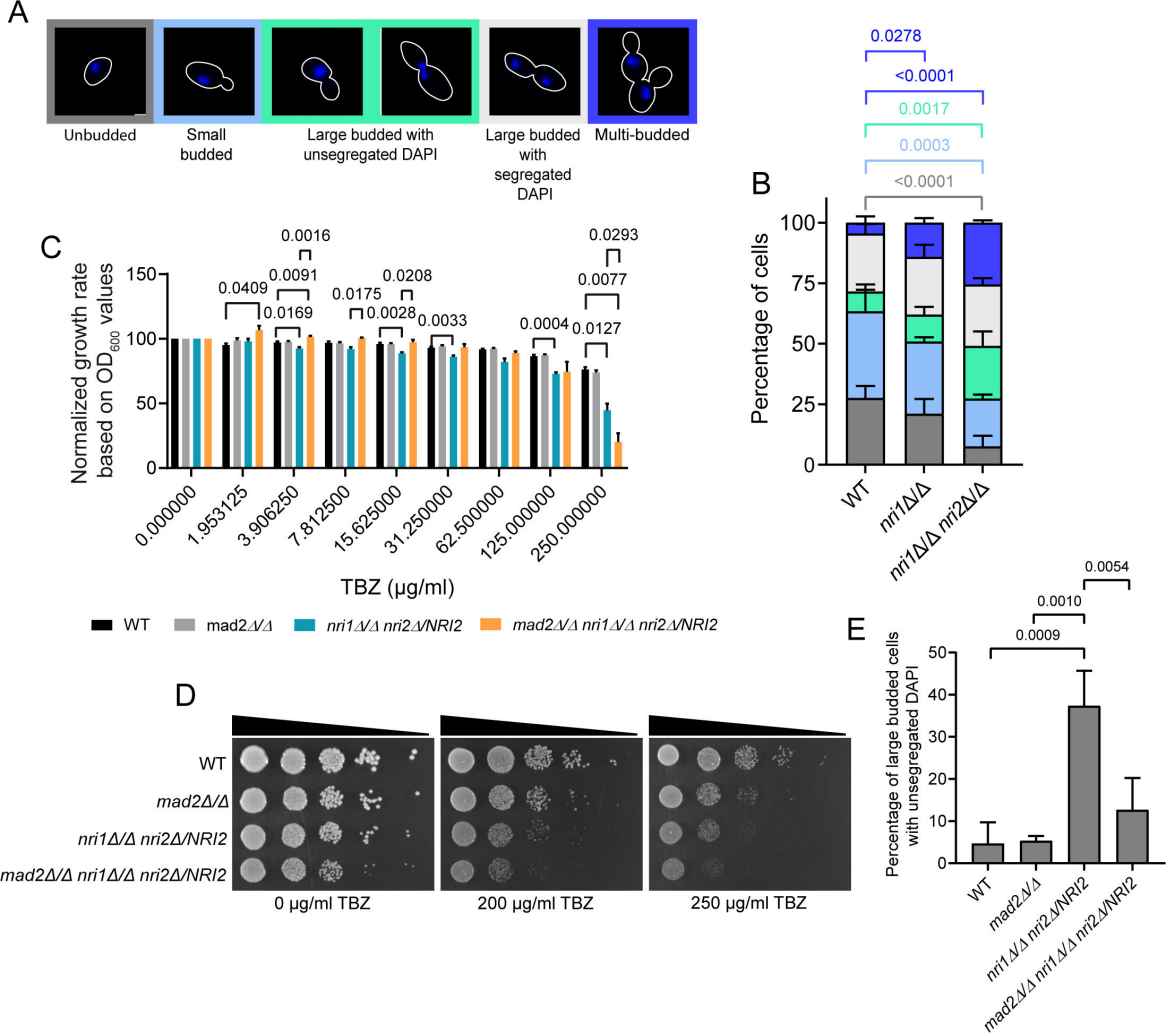
*nri* mutants display defective cell cycle progression. (**A**) Representative images of different cell morphologies across the cell cycle stages. Scale bar 2 µm. (**B**) Stacked bar graph showing the quantification of cell morphologies. *N* = 300. WT, wild type. (**C**) Quantification of TBZ sensitivity by broth microdilution assay based on OD_600_ values. Statistical analysis was performed by two-way ANOVA. (**D**) Analysis of TBZ sensitivity by spotting assay. (**E**) Budding index analysis for indicated strains to assess abrogation of G2/M arrest. Error bars indicate standard deviation. *N* = 150. Data were obtained from three biological replicates. Statistical analysis was performed by one-way ANOVA. A *P*-value of < 0.05 was considered significant. Only significant *P*-values are mentioned in the graphs.

Due to the observed growth defect and DEGs under “cell cycle,” we first determined the cell death frequency in the mutants by staining the log-phase cells with propidium iodide but observed no significant defects in the mutants (Fig. S1D). We then analyzed the budding index in the asynchronously growing log-phase cells to understand the defects the mutants may harbor while progressing through the cell cycle. The behavior of the DAPI-stained nucleus with respect to the bud morphology was used to determine the cell cycle stages (Fig. 3A). We did not observe any defects in the gross nuclear morphology and segregation in the mutants (Fig. 4A; Fig. S1E). However, both the mutants showed a significant increase in the percentage of multi-budded cells, 14.1% in the *nri1Δ/Δ* mutant and 25.5% in the *nri1Δ/Δ nri2Δ/Δ* double mutant, compared to only 4.5% in the WT strain (Fig. 4A). Multi-budded cells (Fig. S2A for a representative differential interference contrast [DIC] microscopy image) might arise from defective cytokinesis/mitotic exit or cell separation defect. To address this, we treated the cells with zymolyase, which removes the cell wall and thus separates the cells with separation defects. Representative DIC image of the multi-budded cell after zymolyase treatment is included in supplementary information (Fig. S2B). We quantified zymolyase activity and observed that although there was a slight difference in the zymolyase activity across the strains, it was not statistically significant (Fig. S2C). However, zymolyase treatment could not change the percentage of multi-budded cells, indicating that the multi-budded cells exhibited a cytokinesis defect rather than a cell separation defect (Fig. S2D).

Notably, a significantly higher percentage of *nri1Δ/Δ nri2Δ/Δ* cells also showed large buds with unsegregated DAPI (21.8%), a phenotype similar to RSC mutants (32, 40, 48), indicating possible arrest at G2/M stage (Fig. 4A). To understand whether this arrest is mediated by the activation of the spindle assembly checkpoint (SAC), *NRI* genes were individually deleted in the *mad2Δ/Δ* strain used earlier (49). Although we could not construct a triple mutant, perhaps due to synthetic lethality, we analyzed the thiabendazole (TBZ) sensitivity of the *mad2Δ/Δ nri1Δ/Δ nri2Δ/NRI2* strain. We performed a broth microdilution experiment (Materials and Methods) to quantify the TBZ sensitivity and normalized it with the inherent growth defect. We observed a synthetic sick phenotype and a significant increase in TBZ sensitivity of the *mad2Δ/Δ nri1Δ/Δ nri2Δ/NRI2* mutant compared to the *nri1Δ/Δ nri2Δ/NRI2* mutant based on OD_600_ values (Fig. 4B). The former mutant showed a slight reduction in the growth rate in the absence of the drugs as well, altogether indicating a genetic interaction between *MAD2* and *NRI1* genes (Fig. 4C). Surprisingly, we could not find any significant difference in the viability of the *mad2Δ/Δ* compared to the viability of the WT strain till 250 µg/mL TBZ concentration. Increased MIC in broth-based assays compared to agar-based assays has been reported for several bacterial strains and antibiotic drug pairs (50). Thus, if we increase the TBZ concentration further in the broth microdilution method, we might observe a drop in viability for the *mad2Δ/Δ* mutant. Furthermore, the saturated culture of *nri1Δ/Δ* mutant displayed a significant increase in the proportion of large budded cells with unsegregated DAPI during budding index analysis, indicating G2/M arrest (data not shown). For this, we observed that the saturated culture of *nri1Δ/Δ nri2Δ/NRI2* mutant also had a significantly increased G2/M-arrested cell population, but the arrest phenotype was alleviated in *mad2Δ/Δ nri1Δ/Δ nri2Δ/NRI2* mutant (Fig. 4D). Overall, these results indicate that SAC contributes to the G2/M arrest phenotype observed in the *nri* mutants.

### *nri* mutants display defects in spindle morphology at the later stage of the cell cycle

**Fig 5:**
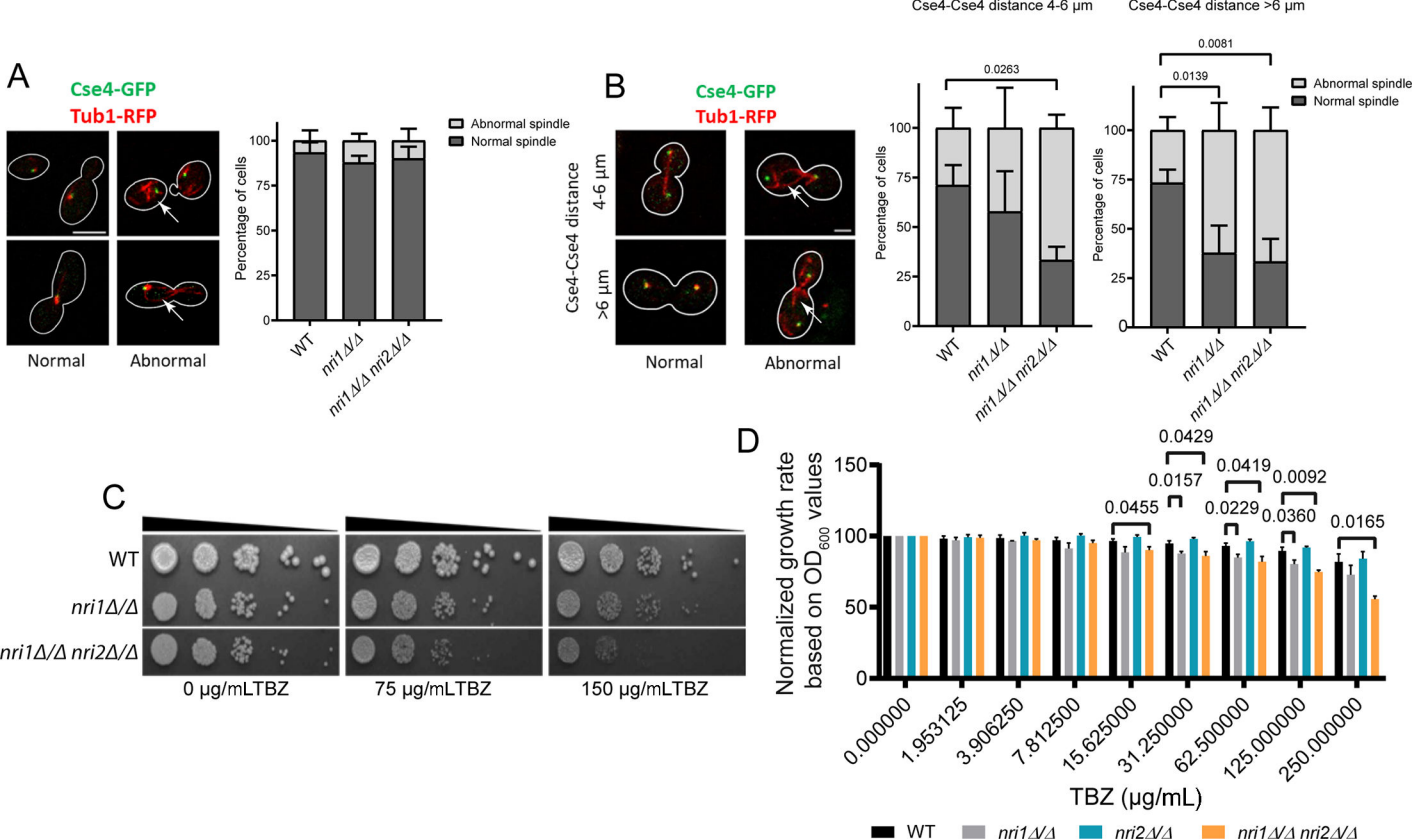
*nri* mutants exhibit abnormal spindle morphology at the later stage of the cell cycle. (**A**) Left: Representative images of normal and abnormal spindle morphologies in the pre-anaphase cells. Scale bar 2 µm. Right: stacked bar graph of the percentage of pre-anaphase cells was plotted. *N* = 90. (**B**) Left: Representative images of normal and abnormal spindle morphologies in post-anaphase cells categorized into two groups based on the Cse4-Cse4 distance. An abnormal spindle is denoted by white arrowheads in **A and B**. Scale bar 2 µm. Middle and right: Stacked bar graphs of the percentage of cells were plotted for both groups. *N* = 45. Statistical analysis was performed by two-way ANOVA. (**C**) Image showing growth of 10-fold serial dilutions of the cells spotted on YPDU plates containing indicated concentrations of TBZ. Plates were incubated at 30°C for 48 h and imaged. (**D**) Quantification of TBZ sensitivity by broth microdilution assay based on OD_600_ values. Statistical analysis was performed by two-way ANOVA. *P*-values of < 0.05 were considered significant. Only significant *P*-values are mentioned in the graphs.

The cell cycle progression defect and genetic interaction with the *mad2Δ/Δ* mutant indicate that microtubule or KT-related defects may exist in the *nri* mutants. Since abnormal spindle morphologies were observed in Sth1-depletion mutant in *C. albicans* (32), we therefore tested the spindle morphology in the *nri* mutants by live cell imaging of the asynchronously growing log-phase cells harboring Cse4-GFP and Tub1-RFP. Cells were categorized into two groups based on the cell cycle stages, namely pre-anaphase and post-anaphase, judged by the bud size and Cse4-GFP signal, respectively, and Tub1-RFP morphology was scored (Materials and Methods). In pre-anaphase cells, the normal spindle morphology was scored when Tub1-RFP and Cse4-GFP signals colocalized and remained proximal to the spindle poles or when the spindle axis (the Tub1-RFP signal joining the poles) was found parallel or in acute angle to the bud axis. Excess or broken Tub1-RFP signal away from the Cse4-GFP signal and spindle axis in perpendicular to the bud axis was considered as the abnormal morphology. The normal spindle morphology in post-anaphase cells implied colocalization of Tub1-RFP and Cse4-GFP signals at the spindle poles and/or a single rod-like signal of Tub1-RFP in-between two Cse4-GFP signals. On the other hand, looped, broken Tub1-RFP signals, often non-overlapping with Cse4-GFP signals, were considered abnormal morphology. For all the tested strains, we did not observe any significant defect in the spindle morphology in pre-anaphase cells having no, small, or large buds (Fig. 5A). The post-anaphase cell population was further categorized into early anaphase and late anaphase based on the pole-to-pole length, which was judged by Cse4-Cse4 distance. In budding yeast, the metaphase spindle of 1.5–2 µm increases in length during anaphase B in two stages. First, the spindle elongates at a faster rate until the spindle length reaches 4–6 µm. In the second stage, the elongation occurs at a reduced rate (51, 52), and the spindle disassembly begins when the spindle reaches 90% of its total length (53). Thus, based on these observations, we categorized 4–6 µm and > 6 µm spindle lengths as early and late anaphase spindles, respectively. We observed a significant increase in the cells with abnormal spindles in both early and late anaphase in *nri1Δ/Δ nri2Δ/Δ* double mutant and only in late anaphase in *nri1Δ/Δ* mutant (Fig. 5B). Despite the presence of abnormal spindles, no significant difference in pole-to-pole length was observed between wild type and the mutants (Fig. S3).

Cells with abnormal KT or microtubule functions exhibit sensitivity to antimitotic drugs (54–56). As we observed spindle defects and cell cycle arrest in the *nri* mutants, we tested the sensitivity of these mutants to TBZ. Cells were spot inoculated on the plates containing 0, 75, or 150 µg/mL TBZ. The *nri1Δ/Δ nri2Δ/Δ* double mutant showed hypersensitivity at both concentrations (Fig. 5C). We performed a broth microdilution experiment to quantify the TBZ sensitivity and observed that the TBZ sensitivity is significantly increased in the mutants after normalization with the growth rates based on OD_600_ values of the respective strains (Fig. 5D).

**Fig 6:**
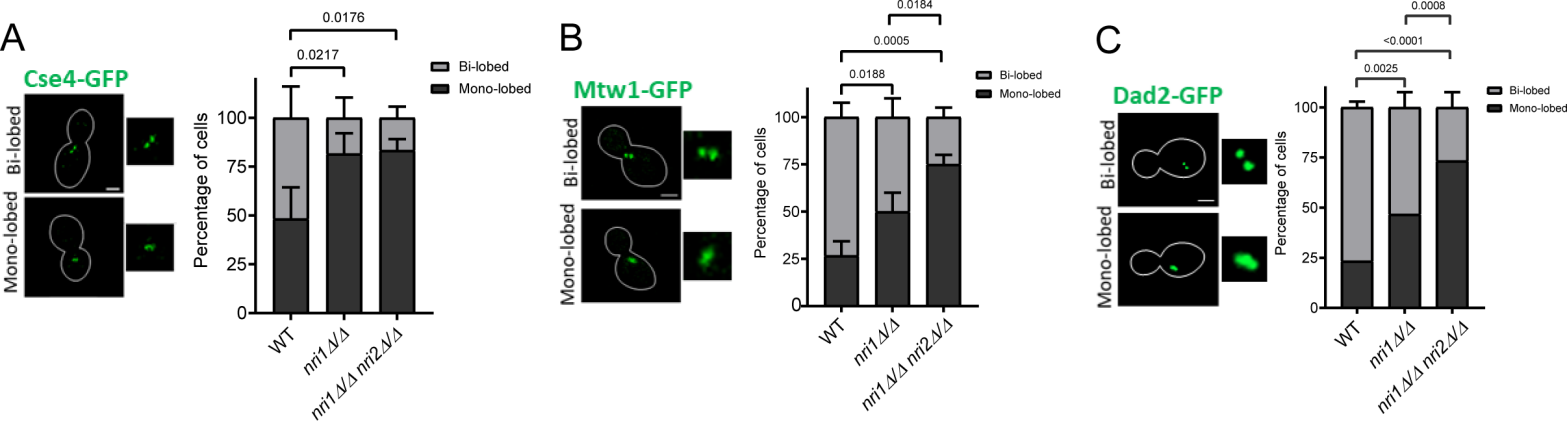
Bi-lobed organization of kinetochore clusters is affected in *nri1Δ/Δ nri2Δ/Δ* double mutant at the G2/M stage. Left: Representative images of the mono-lobed and bi-lobed signal types. Right: Their quantification in G2/M cell population harboring (**A**) Cse4-GFP, (**B**) Mtw1-GFP, and (**C**) Dad2-GFP. Scale bar 2 µm. Statistical analysis was done by one-way ANOVA. Data from three biological replicates plotted with error bars indicating standard deviation. *N* = 30 for each cell cycle stage. A *P*-value of < 0.05 was considered significant. Only significant *P*-values are mentioned in the graphs.

Previous reports suggest a correlation between KT and spindle integrity (49, 57–60). KT integrity can be affected by a defect in an individual KT ensemble. RNA-seq data from the *nri1Δ/Δ nri2Δ/Δ* double mutant revealed a significant reduction in the levels of *NSL1* and *DAD4* transcripts, which code proteins of central (Mtw1 sub-complex) and outer (Dam1 sub-complex) KT (Fig. 3A), respectively (61, 62). As the alteration in the stoichiometry of the KT proteins within the sub-complexes is known to hamper KT integrity in *C. albicans* (63, 64), we measured the intensity of inner (Cse4-GFP), central (Mtw1-GFP), and outer (Dad2-GFP) proteins in the mutants to assess the KT integrity. We did not observe any defect in the integrity of the KT based on the analysis of the GFP intensity of these three fusion proteins (Fig. S4A through F). Interestingly, at the G2/M stage, unlike the wild-type cells with a characteristic bi-lobed GFP signal, a significantly higher population of cells exhibited a mono-lobed GFP signal for all the three KT proteins in the *nri* mutants (Fig. 6A through C). These results indicate that although the KT integrity is not hampered in the *nri* mutants, the disjoining of sister KT clusters due to microtubule-based pulling force to form a bi-lobed organization is largely compromised. This might result from improper pulling force exerted by the microtubules that was found abnormal in the mutants and/or reduced stretchability of the centromere proximal chromatin in the mutants.

### Absence of *NRI* genes does not perturb centromeric cohesion but affects global chromatin

**Fig 7:**
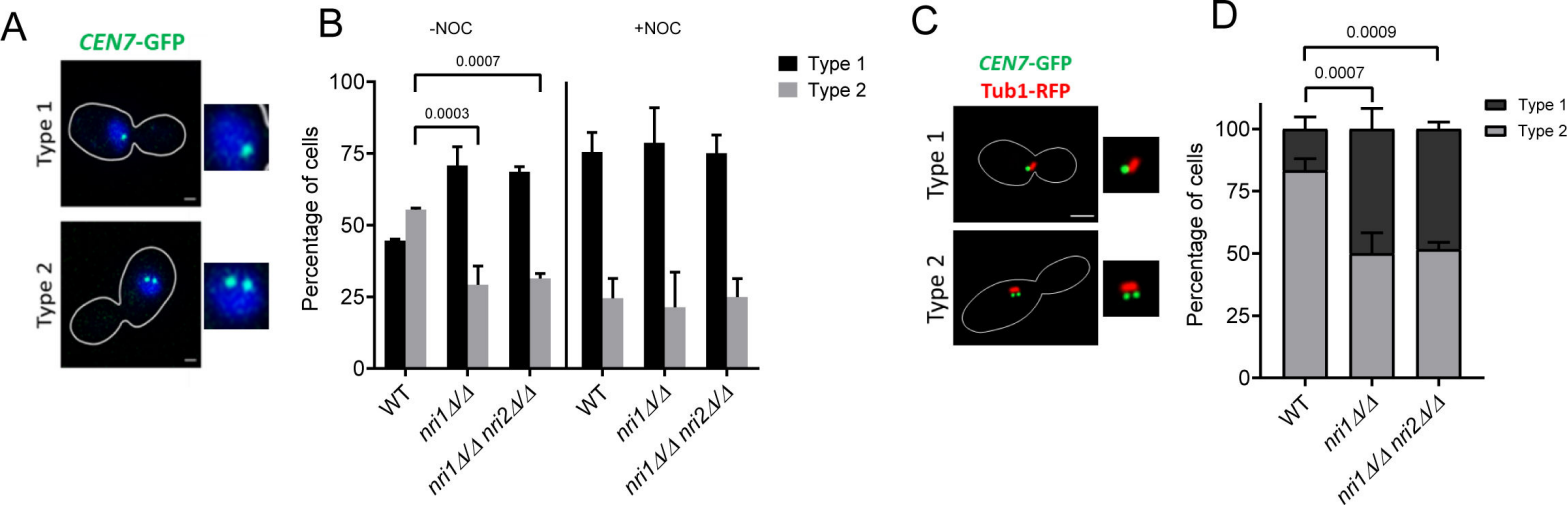
Absence of *nri* proteins does not affect sister chromatid cohesion. (**A**) Representative images of two types of *CEN7*::TetO/TetR-GFP (*CEN7*-GFP) signals. Type 1, single GFP dot depicting cohesed sister chromatids, and Type 2, two GFP dots depicting non-cohesed sister chromatids. Scale bar 2 µm. (**B**) Bar graphs show percentages for the cells of the indicated strains treated with or without NOC. Error bar indicating standard deviation. Statistical analysis performed by two-way ANOVA. Data from three biological replicates. *N* ≥ 99. (**C**) Representative images of *CEN7*-GFP signal categories in metaphase cells showing 0.5–2 µm spindle (Tub1-RFP). Scale bar 2 µm. (**D**) Quantification of the signal categories shown in C in the indicated cells. *N* ≥ 96. Data from three biological replicates. Error bars indicate standard deviation. Statistical analysis performed by one-way ANOVA. A *P*-value of < 0.05 was considered significant. Only significant *P*-values are mentioned in the graphs.

Various reports highlight the function of the RSC complex in establishing and maintaining proper sister chromatid cohesion (38, 39, 65). The correlation of proper cohesion and normal spindle structure is also deciphered in *S. pombe* and mice oocytes (66, 67). Moreover, earlier, we observed that cohesion and spindle-mediated pulling forces determine the resolution of the *CEN7*-GFP signal (68). As we observed abnormal spindle morphology and significantly increased mono-lobed KT-GFP signal in the *nri* mutants, we evaluated the role of Nri proteins in the sister chromatid cohesion. For this, microtubules were depolymerized using 50 µg/mL nocodazole in *CEN7*::TetO-tagged TetR-GFP-expressing strains, and the nucleus was stained using a live cell DAPI staining protocol (68). Depolymerization of the microtubules was confirmed by indirect immunofluorescence using anti-Tub1 antibody. No tubulin signal was observed in around 80% of the nocodazole-treated cells for all the strains (Fig. S5). A GFP signal was observed in large budded cells with unsegregated DAPI to assess the centromeric cohesion in metaphase cells. In the cells with proper chromatid cohesion, two GFP signals from two sister chromatids coalesce into one signal (dot) due to the diffraction limit of the microscope (Fig. 7A, type 1), whereas the signal is visible as two GFP dots (Fig. 7A, type 2) in cells having defective cohesion. We found no significant difference in the type 1 and type 2 signal for the *nri* mutants as compared to the WT strain (Fig. 7B, +NOC), indicating that sister chromatid cohesion is not perturbed in the mutants. In the cells with intact microtubules (-NOC), the sister chromatids experience outward pulling force by microtubules, and because of this, some population of cells harbors two GFP dots (Fig. 7A, type 2). Notably, *nri* mutants displayed significantly less percentage of type 2 signal (Fig. 7B, -NOC). To understand the reason behind a reduction in the type 2 signal, we tagged Tub1-RFP for visualization of the spindles to assess the cell cycle stages and quantified GFP signal only in the cells harboring metaphase spindles (0.5–2 µm length) (Fig. 7C). At metaphase, 83% of the WT cells exhibited type 2 signal. However, 50% and 51%, respectively, of *nri1Δ/Δ* and *nri1Δ/Δ nri2Δ/Δ* mutant cells displayed significant reduction in type 2 signal (Fig. 7D), similar to KT-tagged strains (Fig. 6). This indicated that perhaps the force exerted by the spindles on the KTs in the *nri* mutants is not strong enough to resolve the GFP dots. The observed spindle morphology defects (Fig. 5) in the mutants may be related to this. It was demonstrated that the condensed chromatin behaves as a spring, and the degree of chromatin compaction determines its stiffness (69, 70). Therefore, to test the condensation status of the chromatin, we examined its accessibility to micrococcal nuclease (MNase) in G2/M phase arrested WT and mutant cells and observed that in the mutant, the chromatin is less accessible to MNase than in the wild type, suggesting a more condensed nature of the chromatin in the mutant (Fig. S6).

### Cells without the Nri proteins arrest at S phase

**Fig 8:**
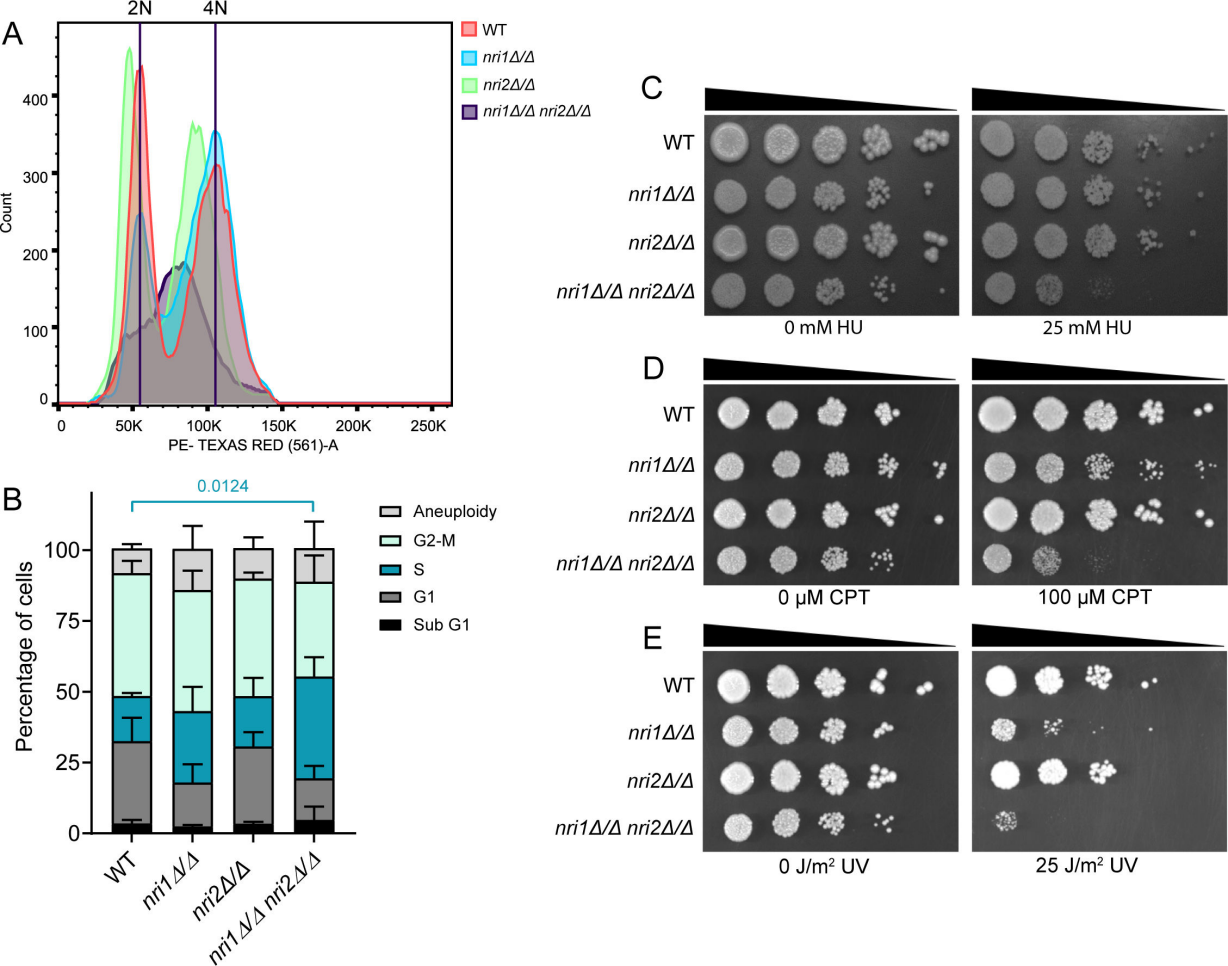
Cells lacking the Nri proteins show S phase arrest and drug sensitivity. (**A**) FACS profiles indicate ploidy of ethanol-fixed, RNase-treated, PI-stained cells. In total, 20,000 events were recorded with BD FACS Aria flow cytometer with PE-Texas Red filter. Gating was done using PI width (X-axis) vs. PI area (Y-axis) to obtain single cell population. After gating, histograms were plotted using PI area (X-axis) for the indicated strains. X-axis indicates PE-Texas Red area (PI intensity) and Y-axis indicates cell count. (**B**) Quantification of the cells in different cell cycle stages based on the gating for PI peak intensity of the WT strain. Data from two biological replicates. Statistical analysis was done by two-way ANOVA. *P*-value of < 0.05 was considered significant. Only significant *P*-values are mentioned in the graph. Sensitivity was analyzed by spotting assay for (**C**) HU, (**D**) CPT, and (**E**) UVC radiation; 10-fold serial dilutions of the cells spotted on YPDU plates containing indicated concentrations of the drugs or UVC radiation dose. Plates were incubated at 30°C for 48 h and imaged.

Although the above results indicate that the *nri* mutants harbor defects in KT-microtubule-related processes and thus arrest at G2/M in SAC-dependent way, the possibility of an additional defect in S phase cannot be ruled out, particularly when replication genes such as *ORC4* and *CDC45* were found as DEGs in the RNA-seq data from the *nri* mutants (Fig. 3A). Since the distinction between the S and G2/M phases using budding index analysis is not accurate as the bud size might increase in case of transient arrest, we used flow cytometry to judge the S phase progression proficiency of the mutant cells compared to the WT. We observed that a significant proportion of the *nri1Δ/Δ nri2Δ/Δ* cells showed S phase arrest (Fig. 8A). For gating, WT peaks were used as a reference for *nri1Δ/Δ* and *nri1Δ/Δ nri2Δ/Δ* mutants. As the *nri2Δ/Δ* mutant showed a leftward shift, its gating was done based on that (Fig. S7). After gating the population, we observed that 15.95%, 25.2%, and 17.8% of the population were in the S phase for the WT, *nri1Δ/Δ*, and *nri2Δ/Δ* mutants, respectively. On the other hand, a higher percentage (36%) of the cell population was in the S phase for the *nri1Δ/Δ nri2Δ/Δ* double mutant. This resulted in a concomitant reduction in the G2/M population in the double mutant. G2/M population decreased to 33.5% as compared to 43.35%, 42.8%, and 41.5% in the WT, *nri1Δ/Δ*, and *nri2Δ/Δ* mutants, respectively. G1 population also reduced to 15.5% and 14.7% in *nri1Δ/Δ* and *nri1Δ/Δ nri2Δ/Δ* mutants as opposed to 29.05% and 27.35% in WT and *nri2Δ/Δ* mutants, respectively. Populations for all other cell cycle stages were comparable in all the strains (Fig. 8B). FACS analysis suggests that at least a fraction of the large budded double mutant cells harboring unsegregated DAPI (from the budding index analysis) might still be in the S phase at the arrested condition, as they may harbor defects in DNA replication. Yeast cells with defective replication exhibit sensitivity to hydroxyurea (HU) and camptothecin (CPT), a topoisomerase I inhibitor (71). We also observed that the *nri1Δ/Δ nri2Δ/Δ* mutant is sensitive to these drugs (Fig. 8C and D), supporting their replication defects and resulting S phase arrest phenotype. We also tested the susceptibility of *nri* mutants to UV radiation. Both *nri1Δ/Δ* and *nri1Δ/Δ nri2Δ/Δ* mutants exhibited hypersensitivity to UV radiation (Fig. 8E). However, we did not observe any sensitivity of *nri* mutants to another DNA-damaging agent methyl methanesulfonate (MMS), indicating a possible involvement of Nri proteins in the regulation of only specific DNA damage repair pathways (Fig. S8). To examine if the S phase arrest of *nri1Δ/Δ nri2Δ/Δ* cells is mediated by the DNA damage checkpoint, we tried constructing *mec1Δ/Δ nri1Δ/Δ nri2Δ/Δ* triple mutant. However, even after repeated attempts, we failed to get true transformants. We also tried deleting both *NRI1* and *NRI2* genes in *dpb3Δ/Δ* cells (72), a mutant of the DNA polymerase epsilon subunit, but got similar results. Thus, we believe that deletion of both *NRI1* and *NRI2* genes causes certain replication defects for which the double mutant shows synthetic lethality either with *mec1Δ/Δ* or *dpb3Δ/Δ* mutation. Thus, we could not verify whether the S phase arrest of *nri1Δ/Δ nri2Δ/Δ* cells is due to the action of the DNA damage checkpoint.

### Nri1 protein has the potential to activate transcription

**Fig 9:**
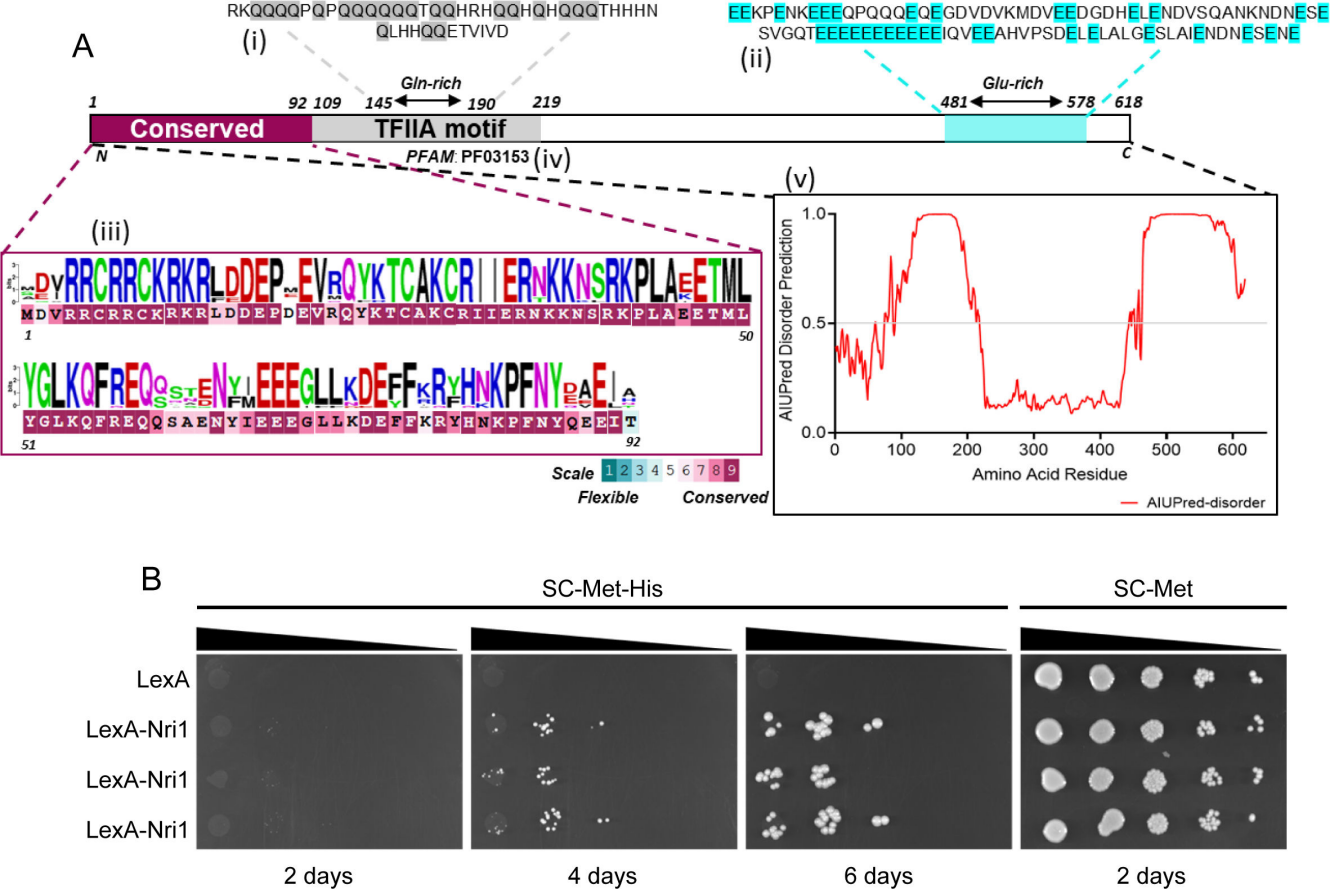
Nri1 protein exhibits a potential transcription activation ability. (**A**) *In silico* analysis revealed various features of the Nri1 protein. PROSITE search detected (i) 145–190 residues to be glutamine-rich (Gln-rich) and (ii) 481–578 residues as glutamic acid-rich (Glu-rich). (iii) ConSurf analysis predicted 1–92 amino acids to be conserved, which displays high similarity with various transcription factors. ConSurf conservation scale ranges from 1 to 9, with the highest conservation colored in magenta. WebLogo was used to make the weighted sequence logo based on the ConSurf multiple sequence alignment. (iv) Motif search using pfam database predicted that 109–219 residues comprise transcription factor IIA (TFIIA) motif. (v) AIUpred predicted 91–217 and 463–618 residues to be disordered. These regions have a disorder prediction score close to 1. A graph is plotted using GraphPad Prism 8.0. (**B**) *Candida* monohybrid assay detected the ability of the SC2H3 strain (5xLexO-*HIS1*) expressing LexA-Nri1, but not LexA alone, to grow on the SC-Met-His plate. Cells from three independent transformants were spotted on the SC-Met-His and SC-Met plates, incubated at 30°C for the indicated time, and imaged.

The transcriptional activation exhibited by the chromatin-remodeling complexes is often shown to be executed by one or more of their constituent proteins (73–78). As the absence of Nri1 extensively affected *C. albicans* transcriptome, we wished to examine if it has transcription activation potential. To assess this, we evaluated sequence features of Nri1 using different bioinformatics tools (Fig. 9A). Using PROSITE, we observed the presence of glutamine- and glutamic acid-rich stretches at the N- and C-terminal regions of Nri1 protein, respectively. Several reports suggest that glutamine-rich motifs play roles in transcription regulation (79–81). Additionally, the glutamic acid-rich regions within intrinsic disorder regions are shown to be involved in transcription activation (82–84). Aligning with this, AIUPred (85) also predicted that the glutamic acid-rich region (residues 463–618) of Nri1 is disordered. Then, a motif search using the Pfam database predicted transcription factor IIA, alpha/beta subunit motif between 109 and 219 amino acids of the Nri1 protein. To understand the evolutionary conservation of the amino acid residues, ConSurf analysis showed the N-terminal region (residues 1–92) is conserved across multiple proteins, including several predicted transcription factors from various *Candida* species (UniProt IDs: A0A8J5QLV9, H8WXN7, A0A8H7ZCI4, G8BF45, A0AAD5BGF6, and A0A642UPY1). On the other hand, no domain/motif was predicted for the Nri2 protein.

We then adapted a CUG codon optimized mono-hybrid system to assess experimentally the transcription activation potential of the Nri1 protein (86). If Nri1 has a transcription activation ability, a fusion protein of Nri1 and LexA DNA binding domain should be able to activate transcription of the reporter gene (*HIS1*) placed downstream of the LexA operator (5XLexO-*HIS1*), making the mono-hybrid strain (SC2H3) histidine prototroph. The LexA-Nri1 fusion protein was functional as it complemented the known stress sensitivity phenotype of the *nri1Δ/Δ* mutant (Fig. S9). We observed that SC2H3 expressing LexA-Nri1, but not LexA alone or LexA-Nri2 (Fig. S10), was able to grow on the histidine dropout plate, supporting the transcriptional activation potential of Nri1 protein (Fig. 9B). However, further experiments are required to understand whether the observed transcription activation is achieved by Nri1 alone or in conjunction with other proteins of the RSC complex.

## DISCUSSION

The RSC chromatin-remodeling complex is a multi-subunit complex that regulates myriad functions in eukaryotes (16). Previous work by our group identified the composition of the *C. albicans* RSC complex and discovered that the complex harbors two novel CTG clade-specific proteins, namely Nri1 and Nri2 (30). As the species-specific subunits regulate various processes across species, in this study, we characterized the roles of the Nri proteins with respect to the cell cycle progression of the organism.

We observed that *NRI1* deletion or combined deletion of *NRI1* and *NRI2* resulted in a marked reduction in the growth rate of *C. albicans*, implying their significance in cell proliferation (Fig. 1A through C). On the other hand, *NRI2* deletion had no apparent growth defect (Fig. 1A; Fig. S1A). Synthetic sick phenotype of the *nri1Δ/Δ nri2Δ/Δ* double mutant indicated that these genes might regulate cell proliferation by promoting different functions—some are partially overlapping, and some are distinct (Fig. 1D). This observed reduction in the growth rate occurred because of disruption in the cell cycle progression and not due to increased cell death (Fig. S1D). Budding index analysis revealed that the cells lacking the Nri proteins tend to spend more time in G2/M and/or in S phase and additionally show cytokinesis defects as judged by increased percentage of multi-budded cells (Fig. 4A; Fig. S2D). In *C. albicans*, impaired cell wall integrity is known to influence cytokinesis (87). Since the sensitivity of the *nri* mutants to cell wall-damaging agents indicates cell wall alteration (Fig. S1B) (88), this can be a possible explanation for the observed defect in cytokinesis.

Since an accumulation of large budded cells with unsegregated DAPI in *nri1Δ/Δ nri2Δ/Δ* cells can account for defects both in G2/M and S phases, we investigated this further. We believe that there is a transient G2/M delay due to SAC activation, as we observed genetic interaction between *NRI1* and *MAD2* genes (Fig. 4B and C). Such a delay was also reported for *rsc* mutants of ATPase subunit (Sth1) in *S. cerevisiae* (40, 89) and in *C. albicans* (32). This argues that the Nri proteins are required for proper KT-microtubule functions. Interestingly, the effect of removing the Nri proteins is somewhat different from removing Sth1 of the RSC Complex. Both *nri* and *sth1* mutants showed microtubule depolymerizing drug sensitivity (Fig. 5D and E; 32, 40), but unlike the *sth1* mutant, *nri* mutants showed no sister chromatid cohesion (Fig. 7A and B; 32, 40) but a massive anaphase spindle defect (Fig. 5B; 32, 40). In budding yeast, RSC is shown to promote the association of cohesin (Mcd1) with chromosomal arms, but not with centromeres (37). Since with the *CEN7*-GFP strain, we could only detect centromeric cohesion, this can be one of the reasons why we did not observe a cohesion defect in the *nri* mutants.

In addition, surprisingly, we noticed non-disjunction of KTs or centromeric chromatin in a sizable fraction of the *nri* mutant cells (Fig. 6, 7C and D), which was not reported for the *sth1* mutant. The observed spindle defect (Fig. 5) may account for this by not providing the requisite pulling force, which needs to be measured. The non-disjunction may also arise due to higher compaction of the chromatin in the mutants (Fig. S6) as the loss of RSC function is known to reduce DNA accessibility and nucleosome crowding-mediated increase in the chromatin compaction (90–92). As the increased chromatin compaction can resist splitting of the fluorescence signals, this might also explain the increased mono-lobed signal of the KTs or *CEN7*-GFP in the *nri* cells (Fig. 6 and 7D; 55). For KT, localization analysis using GFP-tagged KT proteins, cells at the G2/M stage were identified and scored based on the large bud harboring an unsegregated GFP signal within the mother. The observed increase in mono-lobed signal may arise if the cells are in the S phase, particularly as the *nri1Δ/Δ nri2Δ/Δ* double mutant exhibited S-phase arrest (Fig. 8). To address this issue, we labeled the spindle using Tub1-RFP and analyzed only those cells harboring ~2 µm metaphase spindle for mono-/bi-lobed pattern of localization of sister *CEN7*s marked with GFP (Fig. 7C). A similar observation in both sets of these experiments (Fig. 6, 7C and D) confirms that the cells analyzed using GFP-tagged KT proteins were indeed at the G2/M stage. However, we observed a slight variation in the mono- and bi-lobed percentages in *nri1Δ/Δ* and *nri1Δ/Δ nri2Δ/Δ* mutants for *CEN7*-GFP, Cse4-GFP, Mtw1-GFP, and Dad2-GFP signals. The difference in spatial distances of these signals from the centromere, thus experiencing different microtubule pulling force, may account for this variation. It was demonstrated that the condensed chromatin behaves as a spring during nuclear division (69, 70) and the degree of chromatin compaction regulates its stiffness (93). The mitotic spindle behaves as a beam with limited mechanical strength/load, beyond which it bends/buckles (94). In mammalian cells, during late telophase, the microtubules depolymerize or break at the point of microtubule bending (95). Thus, it is possible that in the *nri* mutants at metaphase, the spindle force cannot sufficiently stretch the higher compacted chromatin, leading to unresolved (mono-lobed) centromere/KT signal. Consequently, upon anaphase onset, a larger force perhaps is required to pull the chromosomes toward the poles, which may lead to the observed abnormality in the spindle (Fig. 5). The malfunctioning of the kinesin motors and the microtubule-associated proteins acting at the spindle midzone during anaphase also cannot be excluded. We also observed that the *nri1Δ/Δ nri2Δ/Δ* cells are impaired in timely completing the S phase using FACS analysis (Fig. 8A and B), which is also reflected by their HU and CPT sensitivity (Fig. 8C and D). Observed UV radiation sensitivity also indicated the possible roles of Nri proteins in the regulation of DNA damage repair mechanisms. This is not surprising, given the fact that RSC, through nucleosome repositioning, facilitates replication by promoting repair of intrinsic DNA damage generated during replication (34, 35, 96). For the *nri2Δ/Δ* mutant, FACS peaks showed overall leftward shift, without altering cell cycle progression similar to *S. cerevisiae orc4* mutant (97). However, the role of *nri2Δ/Δ* in ploidy maintenance needs to be studied further.

Chromosome missegregation is a hallmark outcome of perturbation in chromatin, KT, or microtubule spindle from yeast to humans (98, 99). However, surprisingly, despite cell cycle progression defects and alterations in spindle morphology, KT, and chromatin elasticity, we did not observe any gross chromosomal segregation defects in the *nri* mutants (Fig. S1E). This is a remarkable divergence from frequently reported phenotypes for the mutants with defects in centromere, KT, or spindle (58, 100–104). The absence of such missegregation here might suggest that although cell cycle progression is perturbed in *nri* mutants, salvage mechanisms still sustain fundamental chromosome segregation fidelity and viability, at least under the examined conditions. Further study of cell cycle-dependent dynamics of chromosome segregation, along with spindle behavior in the *nri* mutants, might provide mechanistic insights into how *C. albicans* can tolerate spindle and chromatin defects, potentially relevant for the adaptability and pathogenicity of the organism.

In summary, our results indicate that Nri proteins regulate *C. albicans* proliferation by controlling cell cycle progression at multiple stages. However, we cannot comment on whether Nri proteins perform any of their functions independently of the RSC complex. Additionally, the spectrum of DEGs belonging to a broad range of processes indicates that the loss of Nri1 and Nri2 has widespread consequences (Fig. 2 and 3), likely influencing various cellular pathways beyond the tested phenotypes in this study. The predicted TFIIA motif in Nri1 protein and evidence of LexA-Nri1 fusion protein activating transcription indicate potential direct and indirect roles of Nri1 in the regulation of gene expression. Notably, the presence of DEGs involved in the virulence-related biological processes also points toward the possible role of Nri proteins in *C. albicans* pathogenesis. As fungal fitness also affects its virulence, the potential role of Nri proteins, mainly Nri1, in the regulation of *C. albicans* virulence cannot be denied. Sensitivity of *nri* mutants to physiologically relevant stressors also supports this hypothesis (Fig. S1B). Hence, a separate study has been undertaken to delineate the pathogenic potential of the *nri* mutants. Furthermore, given the CTG clade-specific nature of the Nri proteins, a structural characterization of these proteins will be essential to understand how they function within the *C. albicans* RSC complex, and such information will be instrumental to explore, in the future, the potential of the Nri proteins as novel anti-*Candida* drug targets.

## MATERIALS AND METHODS

### Strains, growth conditions, and transformation

*C. albicans* strains and plasmids used in this study are mentioned in Tables S1 and S2. Primers used for strain construction and validation are mentioned in Table S3.

All the *C. albicans* strains were grown in YPDU (1% yeast extract, 2% peptone, and 2% dextrose, supplemented with 100 µg/mL uridine) medium at 30°C, unless stated otherwise. The lithium acetate transformation protocol was used to construct *C. albicans* strains (105). For the selection of transformants, YPDU + 100 µg/mL nourseothricin or synthetic media without appropriate amino acids was used.

### Growth rate analysis

Overnight grown *C. albicans* culture was used to set an OD_600_ of YPDU media to 0.15 and grown at 30°C, 200 rpm. Thereafter, OD_600_ was measured every 1 h until the culture reached saturation (15–16 h). Doubling time was calculated from the exponential growth phase.

### Spot dilution assay

In total, 10-fold serial dilutions of log phase WT and mutant strains were spot-inoculated in descending cell concentration based on the experiment: YPDU plate to evaluate growth defect at standard growth conditions, and YPDU containing indicated concentrations of NOC and TBZ. Plates were incubated at 30°C unless stated otherwise, and images were captured 1–2 days post-inoculation.

### Quantification of TBZ sensitivity by broth microdilution

In a 96-well plate, TBZ was serially diluted in 100 µL YPDU, with the concentration ranging from 250 µg/mL to 1.95 µg/mL; 10^5^ *C. albicans* cells from overnight grown culture were inoculated in the 96-well plate in duplicates. The plate was incubated at 30°C for 24 h. After incubation, the OD_600_ of the cultures was measured using the Agilent Epoch2 plate reader. OD_600_ value from “no cell” control was subtracted from OD_600_ values obtained from different concentrations of TBZ, and the resulting values were then normalized with the OD_600_ values obtained from the cultures of the respective strain treated with 0 µg/mL TBZ (growth control). Growth defect in the presence of TBZ was quantified by normalizing the OD_600_ with the OD_600_ of the respective growth control and plotted. The experiment was repeated with three biological experiments.

### Budding index analysis

Log phase cells were harvested and washed with 0.1 M phosphate buffer, pH 7.5 and permeabilized with 70% ethanol. The cells were again washed once with 0.1 M phosphate buffer, pH 7.5, and resuspended in 100 μL of 2 μg/mL DAPI solution. The tubes were incubated in the dark for 20 min, and the images were acquired using a Zeiss Axio Observer Z1 microscope. Cells were classified based on the nuclear position and bud size.

### Zymolyase assay

Log phase cells were washed and resuspended in spheroplasting buffer. Cells were treated with 0.1 mg/mL zymolyase T20 (MP Biomedicals) for 15 min, washed, and observed under the Zeiss Axio Observer Z1. The percentage of multi-budded cells was quantified for untreated and treated cells. To test the efficiency of the zymolyase activity, log-phase cells were washed and resuspended in sorbitol buffer to a concentration of 1 OD_600_/100 µL of buffer. The OD_600_ was measured by diluting the solution with distilled water in a 1:10 ratio for each strain, following which 12 µL of 14.2 M β-ME and 6 µL of 10 mg/mL zymolyase T20 (0 min) were added. The zymolyase treatment was carried out at 30°C for 1 h. OD_600_ of the treated culture was measured (with 1:10 dilution in distilled water) at 15-min intervals. The drop in the OD_600_, which is directly proportional to the zymolyase activity, was normalized with the initial 0 min OD_600_ value for each strain. The graph was plotted for percentage zymolyase activity, and statistical analysis was performed by two-way ANOVA.

### DNA ploidy analysis

Flow cytometric analysis was performed according to a previous study (106) with slight modifications. Briefly, 2 × 10^8^ log phase cells were harvested by centrifugation and washed once with 10 mL distilled water. The pellet was then resuspended in 100 µL D/W. Cells were fixed with 70% ethanol for 1 h at RT in a tube rotator. Fixed cells were once washed with PBS and rehydrated by incubating in 1 mL PBS at 4°C for 2 h. Rehydrated cells were then treated with 10 µg/mL RNase for 4 h at 37°C. Followed by RNase treatment, cells were washed with PBS and overnight incubated at 4°C. For propidium iodide staining, cells were incubated with 5 µg/mL PI solution at RT for 30 min in dark. PI staining was assessed by a Zeiss Axio Observer epifluorescence microscope. Cells were then diluted in a ratio of 1:4, vortexed, and sonicated with a probe sonicator for 10 s at 20% amplitude and immediately run in BD FACSAria Fusion flow cytometer with PE-Texas Red filter. Analysis was done using FlowJo software version 10.6.1 according to a previous study (107). Parameters of detailed analysis are mentioned in the respective figure legends.

### Fluorescence imaging

Overnight-grown cultures of fluorescently tagged WT and mutant strains were used to set the OD_600_ to 0.2 in fresh YPDU medium, and the cells were grown till OD_600_ 0.8–1. Cells were washed with 0.1 M phosphate buffer. Cse4-GFP and Mtw1-GFP samples were imaged with a Zeiss L780 confocal microscope. Dad2-GFP samples were imaged with a Nikon Confocal AXR microscope. Images were processed using Zeiss ZEN 3.1 software. First, the chosen Z-stacks were extracted for one channel, and maximum intensity projection (MIP) was performed. The same was followed next for the second channel and then the same for the DIC image. At the end, all the MIP images were merged. Intensity measurement (for GFP-tagged Cse4, Mtw1, and Dad2 strains) was done using the “Profile” tool of Zeiss ZEN 3.1 software. With this tool, a line of the specific size was drawn through the center of the fluorescence signal. As expected, the intensity of the single dot obtained from a mono-lobed signal was more than when it was from a bi-lobed signal, as in the former case, it arose from two non-disjoined sister KT clusters. On the other hand, intensity from both the dots was included in the analysis for the bi-lobed signal. Thus, the signal intensity from both the KTs was included in the analysis irrespective of the signal type.

### Sister chromatid cohesion (SCC) assay

SCC assay was performed according to a previous study (68) with minor changes. Live cell DAPI staining protocol was followed to grow the cells till mid-log phase. At OD_600_ 0.4–0.5, the culture was divided into two parts. Nocodazole to the final concentration of 50 µg/mL was added to one part, and an equal volume of nocodazole solvent DMSO (dimethyl sulfoxide) was added to another part. Cells were grown at standard growth conditions for 2 h, washed twice with 0.1 M phosphate buffer, and imaged. Indirect immunofluorescence for tubulin using anti-tubulin antibody (clone YOL1/34, Bio-Rad) was done to confirm microtubule depolymerization by nocodazole.

### MNase assay

MNase assay was performed according to a previous study (108). Briefly, 10 OD_600_ log phase cells were fixed with 1% formaldehyde for 30 min at 25°C, 100 rpm; 125 mM glycine was added to quench the formaldehyde, and the cells were incubated for 5 min at 25°C, 100 rpm. Fixed cells were then washed and resuspended in 1 mL spheroplast solution containing 100 µg/mL zymolyase. The solution was incubated at 30°C until 90% of the cells were spheroplasted. Spheroplasts were washed and resuspended in 2 mL MNase digestion buffer; 30 µL MNase was added, and the solution was incubated at 37°C for 0, 15, 30, and 40 min, respectively. The reaction was stopped by adding 150 µL stop solution. RNase treatment was conducted at 37°C for 30 min. Proteinase K treatment was performed at 65°C by overnight incubation. Chromatin was purified by phenol:chloroform:isoamyl alcohol, followed by ethanol precipitation. Purified chromatin was dissolved in 50 µL NFW; 5 µL of this was run on 2% agarose gel. The image was quantified with ImageLab software. The band intensity was normalized with the respective lane intensity and plotted. Statistical analysis was performed with two-way ANOVA.

### RNA isolation

Total RNA was isolated from 4 × 10^7^ log phase cells. Cells were lysed by bead beating in 1 mL of RiboEx solution (GeneAll Biotechnology, South Korea). Lysate was centrifuged at 10,000 rpm for 1 min, and the supernatant was transferred to fresh microcentrifuge tubes. In total, 200 µL chloroform was added and mixed by inverting. The sample was incubated at RT for 2 min and centrifuged at 10,000 rpm for 15 min at 4°C. The aqueous layer was transferred to a fresh tube, and an equal volume of isopropanol was added. Samples were incubated at −80°C for 1 h and centrifuged at 12,000 × *g* for 15 min at 4°C. Supernatant was discarded; the pellet was washed with 75% ethanol and air-dried for 10 min. The pellet was resuspended in 50 µL nuclease-free water. DNase treatment was done at 37°C for 30 min to remove any gDNA contamination. Followed by that, RNA was purified using RiboEx solution and chloroform and precipitated using isopropanol. After washing with 75% ethanol and air drying, the RNA pellet was finally resuspended in 30 µL NFW. RNA was quantified using a NanoDrop microvolume spectrophotometer, and integrity was assessed by running 1 µg RNA on the agarose gel. PCR of 500 ng RNA template using *ACT1* qPCR primers was done to confirm the absence of gDNA in the samples. Isolated RNA was stored at −80°C until further use.

### RNA-seq library preparation and sequencing

Before library preparation, isolated RNA was quantified using Qubit fluorimeter, and the RIN value was estimated by Agilent TapeStation. RNA-seq libraries were prepared in-house using the Illumina TruSeq Stranded Total RNA kit with 500 ng of total RNA (Illumina protocol 1000000040499 v00). Prepared libraries were quantified by Qubit fluorimeter and Agilent TapeStation. The library sizes ranged from 238 to 305 bp; 80-bp single-end sequencing of the libraries was done using the Illumina NextSeq550 system, generating approximately 36–52 million reads per sample across four lanes (L001, L002, L003, and L004) for each sample.

### RNA-seq data analysis

The fastq files generated from each lane were processed independently. The fastq files containing the raw reads from RNA-seq were checked for quality using FastQC v0.12.1 (109; http://www.bioinformatics.babraham.ac.uk/projects/fastqc). Reads were trimmed using Trimmomatic v0.39 (110), with the following parameters: ILLUMINACLIP:TruSeq3-SE.fa:2:30:10, LEADING:3, TRAILING:3, SLIDINGWINDOW:4:15, and MINLEN:36. The trimmed reads were mapped to the *C. albicans* SC5314, assembly 22 (A22) reference genome using HISAT2 v2.2.1 (111) using default parameters. For aligning RNA-seq reads, we used a modified FASTA file containing DNA sequences of one set (haplotype A) of homologous chromosomes (Ca22chr1A-7A, chrRA) and chrM from the phased, diploid A22 genome assembly of *C. albicans* SC5314 version_A22-s07-m01-r198_chromosomes.fasta (downloaded on 07-01-2024 from CGD). The resultant BAM file was used along with the GFF file (C_albicans_SC5314_version_A22-s07-m01r198_features_with_chromosome_sequences.gff) downloaded on 07-01-2024 from CGD to obtain the count matrix containing the raw counts of reads mapped to haplotype A and mitochondrial transcripts for each sample using the Subread package (featureCounts v2.0.6) (112). The “-g Parent” parameter was specifically set to count the reads mapped to the transcripts in the GFF file. For differential gene expression analysis, the raw read counts from the four lanes corresponding to each sample were first combined (Data S3) and then analyzed using the DESeq2 v1.42.1 package (113) in R v4.3.3. Differentially expressed genes (DEGs) were defined using a log2 Fold Change cutoff of >1 or <−1 and an FDR (adjusted *P*-value) of <0.05. The associated data for *nri1Δ/Δ* single mutant and *nri1Δ/Δ nri2Δ/Δ* double mutant w.r.t. WTs are provided in the Supplementary information (Data S1 to S3). CGD GO Slim Mapper tool was used for Gene Ontology analysis.

### Enrichment analysis using GSEA

GSEA (114) was performed using genes obtained from DESeq2 analysis of the RNA-seq data. The *C. albicans* Assembly 22 gene IDs of DESeq2 output were converted to probe IDs using Candida.chip (downloaded from http://www.candidagenome.org/download/community/GSEA_Nantel_2012/) and ORF19_Assembly22_mapping.tab (downloaded from CGD) files. These probe IDs were assigned a rank score based on the formula −log_10_(*P-*value) * sign(log_2_FoldChange). Rows with empty or NA probe IDs and/or rank scores were removed from further analysis. The resulting rank file (.rnk) was imported into GSEA v4.3.3, in addition to the AS_UdeM_gene_sets_V2_2024.gmt file, kindly provided by Dr. Adnane Sellam, McGill University (personal communication), containing the probe IDs under each gene set. The GSEA analysis parameters used were as follows: scoring_scheme, weighted; set_max, 2000; set_min, 5; nperm, 100; collapse, No_Collapse; and norm, meandiv; with all other parameters set to their default values. Gene sets were organized into a network using EnrichmentMap v3.5.0 (115) and visualized using Cytoscape v3.10.3 with default parameters.

### *In silico* analysis of Nri proteins

The protein sequences of Nri1/2 were obtained from the CGD. Furthermore, these protein sequences were used for the detection of protein domain/motif/family using Motif Search (https://www.genome.jp/tools/motif/) that scans them against a collection of motifs in the Pfam database (116), as well as using PROSITE Scanning (117). Furthermore, the ConSurf web server (118) was used to identify functionally important and conserved regions in the proteins. The proteins obtained from the ConSurf server were further aligned using the CLUSTALW (119), and the weighted sequence logo was obtained using the WebLogo server (120).

## Data Availability

Output files for RNA-seq analysis and GSEA are uploaded as supplementary information (Data S1to S5). RNA-seq raw data has been deposited to the NCBI Gene Expression Omnibus (GEO) with the accession number GSE325007.

## SUPPLEMENTAL MATERIAL

Data S1 msphere.00084-26-s0001.csv DESeq2 output for *nri1* vs. WT comparison.

Data S2 msphere.00084-26-s0002.csv DESeq2 output for *nri1 nri2* vs. WT comparison.

Data S3 msphere.00084-26-s0003.csv Sum counts for the RNA-seq data.

Data S4 msphere.00084-26-s0004.xlsx GSEA results for *nri1* mutant.

Data S5 msphere.00084-26-s0005.xlsx GSEA results for *nri1 nri2* mutant.

Supplemental information msphere.00084-26-s0006.pdf Supplemental figures and tables.

ASM does not own the copyrights to Supplemental Material that may be linked to, or accessed through, an article. The authors have granted ASM a non-exclusive, world-wide license to publish the Supplemental Material files. Please contact the corresponding author directly for reuse.
