# Supplementary material for: CTG clade-specific proteins of the RSC chromatin-remodeling complex regulate cell cycle progression of a critical priority fungal pathogen, Candida albicans
Source: mSphere. 2026 Mar 30;11(4):e00084-26. doi: 10.1128/msphere.00084-26 (PMC13123710; doi:10.1128/msphere.00084-26)
Supplement: Supplemental information — Supplemental figures and tables. [file msphere.00084-26-s0006.pdf]

Figure S1

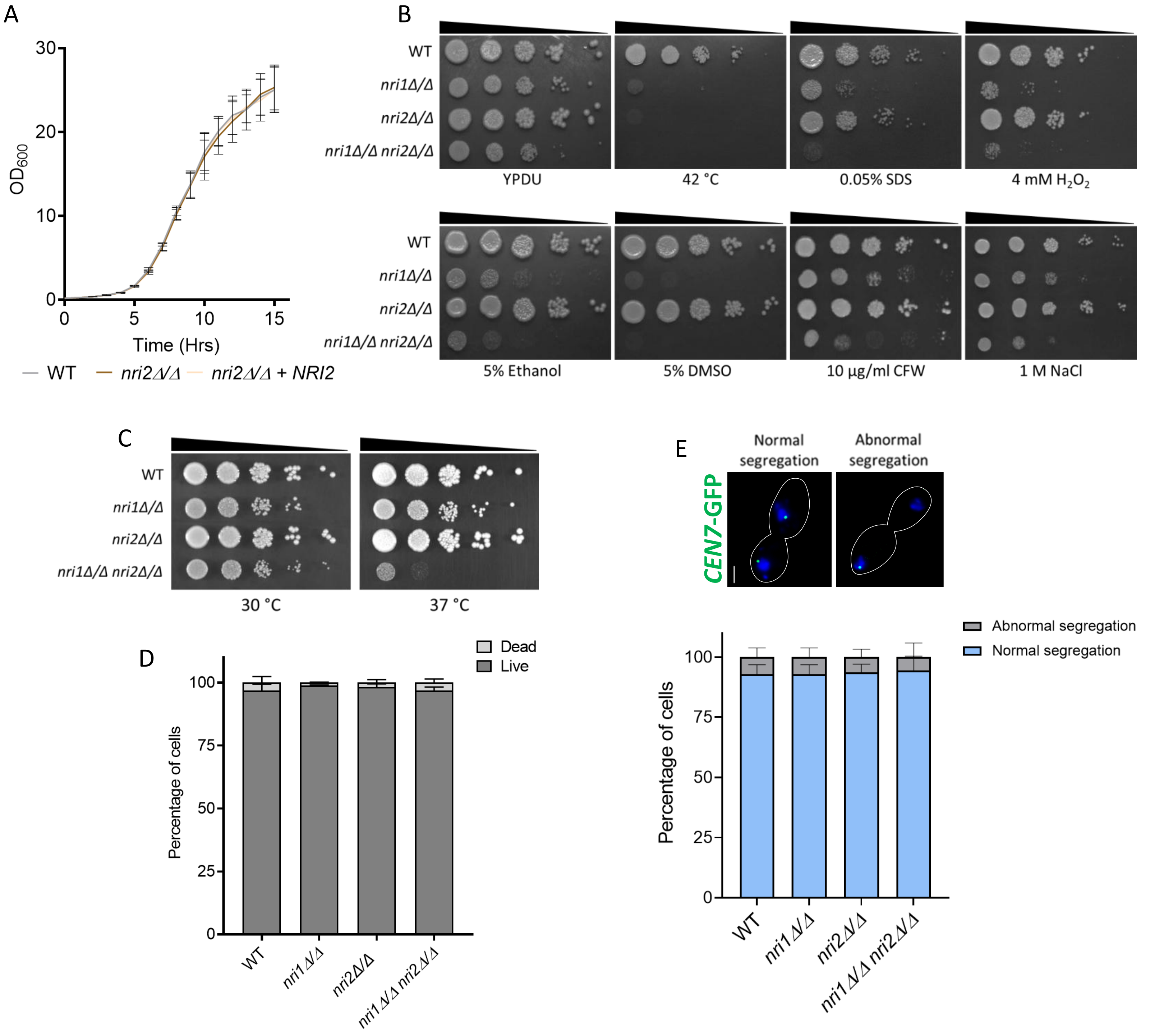

Figure S1: Growth analysis of *nri* mutants.
A. The growth curve for WT, *nri2*Δ/Δ mutant, and *nri2*Δ/Δ +*NRI2* re-integrant strain measured at standard growth conditions. OD_{600} was recorded every 60 mins. Mean values with standard deviation from three independent experiments were plotted. **B, C .** Image showing growth of 10-fold serially diluted cells spotted on the YPDU plate containing indicated stress inducing agent. Plates were incubated at 30 °C, unless stated otherwise, for 48 hrs and imaged. **D.** Live and dead cells for the indicated strains were quantified after staining the log phase cells with 5 µg/ml propidium iodide. N = 750. Data are from three independent experiments. Error bars indicate standard deviation. **E.** Representative images of normal and abnormal *CEN7*-GFP segregation patterns. Scale bar 2 µm. Bar graph represents quantitation of the patterns in the indicated strains. Error bars indicate standard deviation. Data are from three biological replicates. N ≥ 145. Statistical analysis performed by one-way ANOVA. p-value of < 0.05 was considered significant. Only significant p-values are mentioned in the graphs.

Figure S2

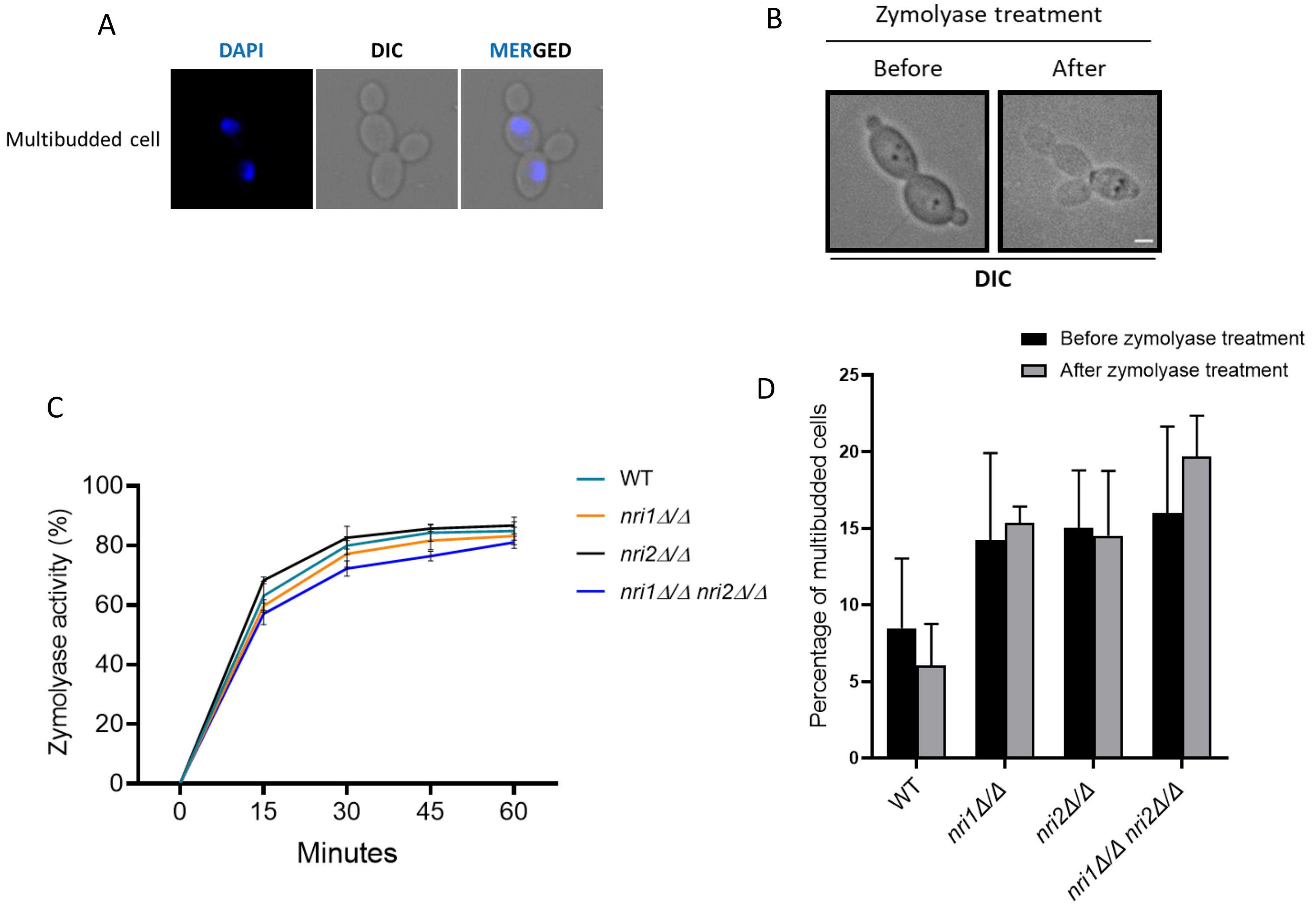

Figure S2: *nri* mutants exhibit cytokinesis defect.
Representative DIC image of the multi-budded cells from **A.** budding index analysis and **B.** before and after zymolyase treatment. **C.** Quantification of zmolyase efficiency as mentioned in the text. Statistical analysis was performed by two-way ANOVA. **D.** Log phase cells of the indicated strains were treated with 10 µg/ml zymolyase for 15 min at 30°C. Number of multi-budded cells before or after zymolyase treatment was quantified. N = 300. Data from three independent experiments. Error bars indicate standard deviation. Statistical analysis was performed by one-way ANOVA. p-value of < 0.05 was considered significant. Only significant p-values are mentioned in the graphs.

Figure S3

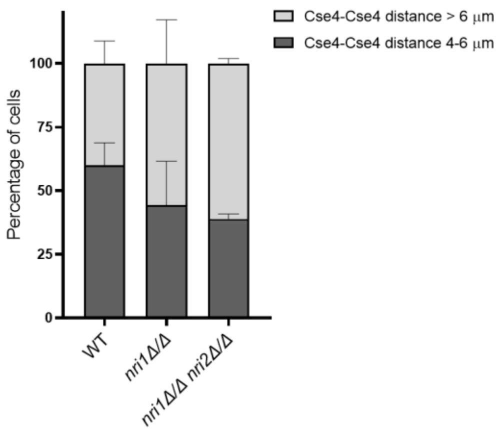

Figure S3: Cse4-Cse4 distance is not altered in *nri* mutants.
Stacked bar graph of the percentage of post-anaphase cells showing indicated Cse4-Cse4 distance was plotted. N = 90. Data from three independent experiments. Error bars indicate standard deviation. Statistical analysis was performed by one-way ANOVA. p-value of < 0.05 was considered significant. Only significant p-values are mentioned in the graph.

Figure S4

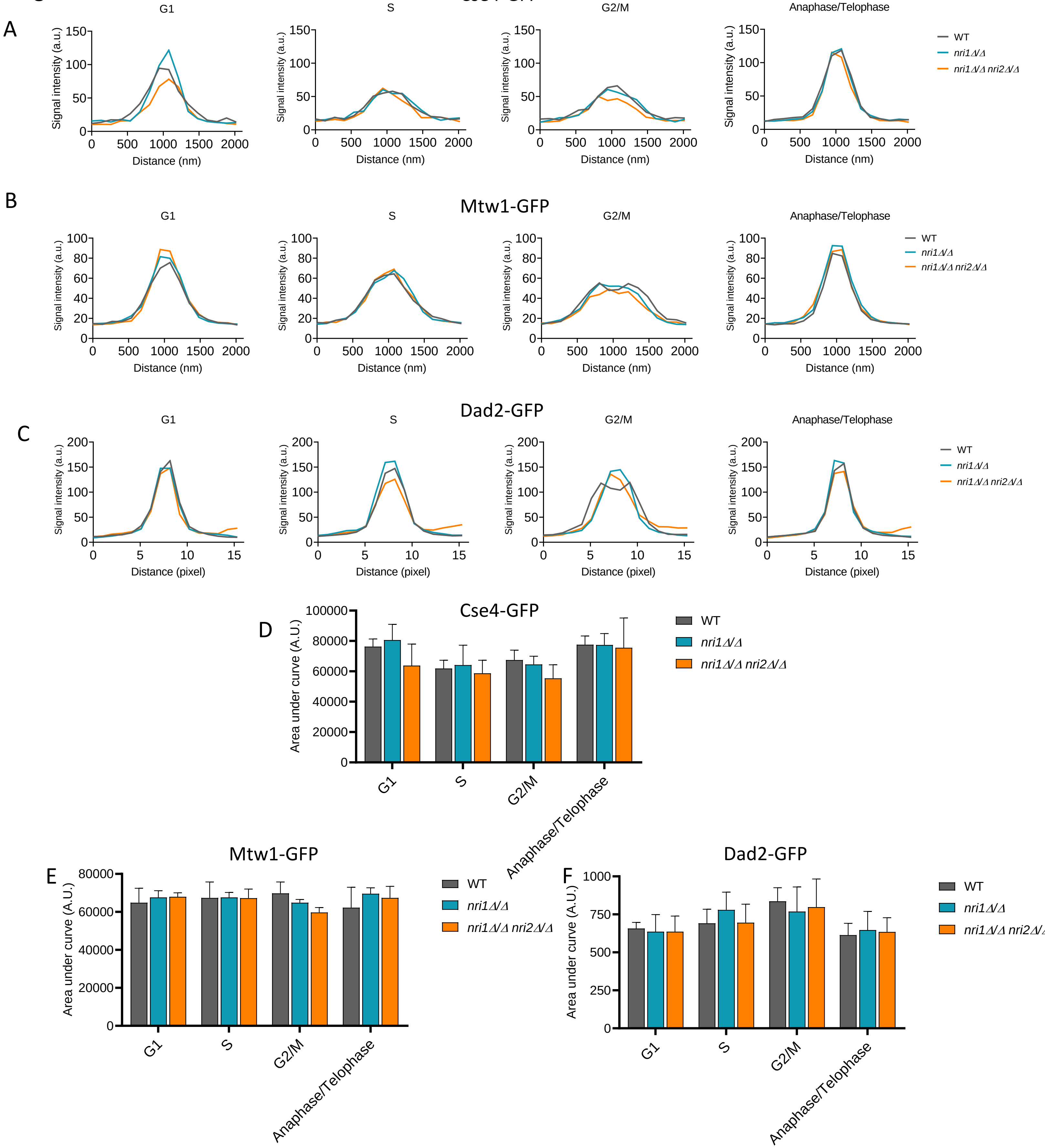

Figure S4: Kinetochore integrity is not altered in *nri* mutants.
The average signal intensity plotted as curves for **A.** Cse4-GFP, **B.** Mtw1-GFP, and **C.** Dad2-GFP. The areas under the curves plotted as bar graphs for **D.** Cse4-GFP, **E.** Mtw1-GFP, and **F.** Dad2-GFP. Statistical analysis was done by two-way ANOVA. Data from three biological replicates are plotted with error bars indicating standard deviation. N = 30 for each cell cycle stage. p-value of < 0.05 was considered significant. Only significant p-values are mentioned in the graph.

Figure S5

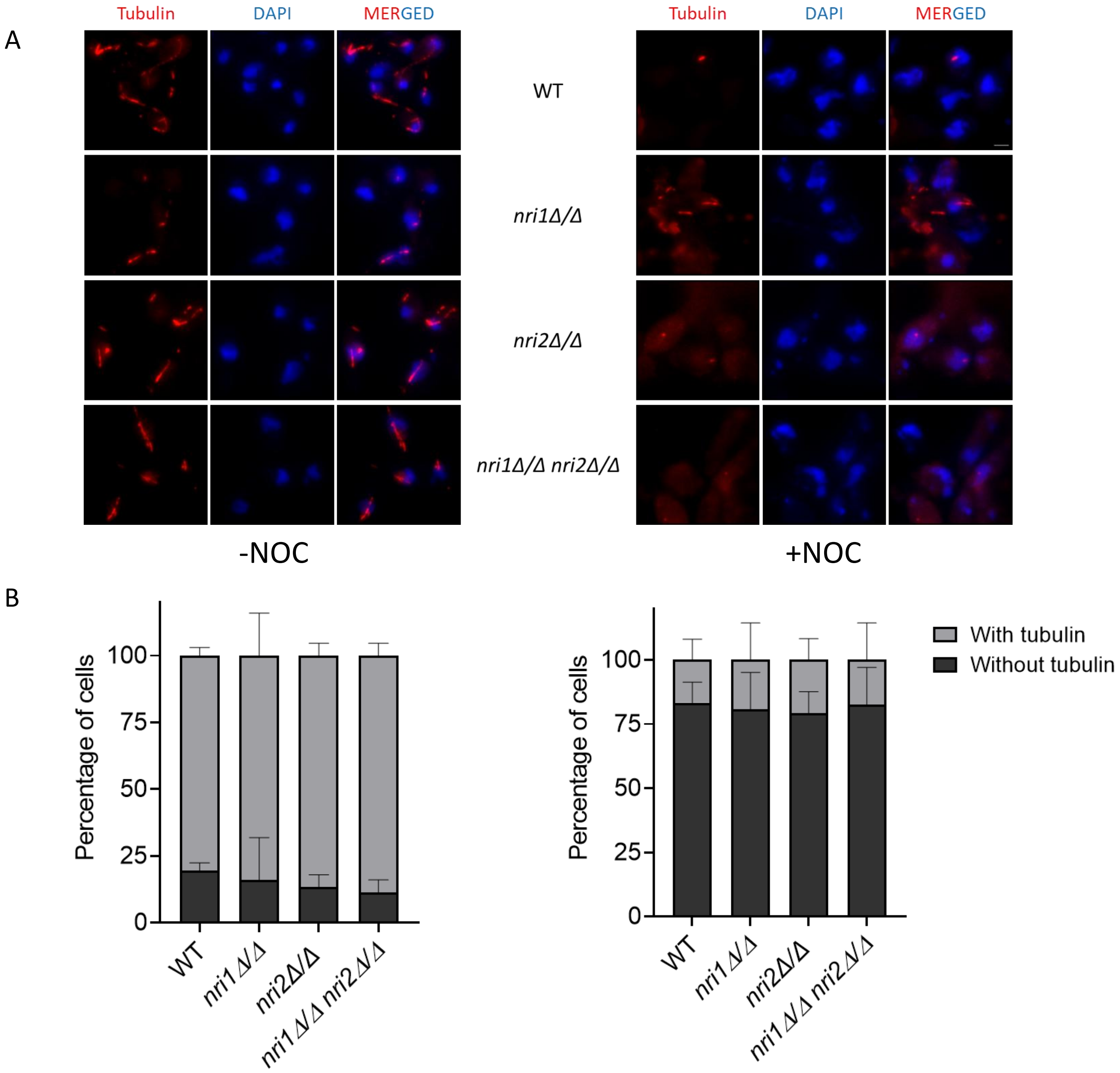

Figure S5: Nocodazole treatment depolymerized microtubules in majority of the cells.
A. Representative images of the cells of the indicated strains showing status of the microtubules visualized using anti-Tub1 antibody through indirect immunofluorescence assay. Scale bar 2 µm. **B.** Percentage of cells with or without tubulin staining for each strain is represented with a stacked bar graph. Experiment was performed in triplicate. Error bars indicate standard deviation. N = 150. Statistical analysis performed by one-way ANOVA. p-value of < 0.05 was considered significant. Only significant p-values are mentioned in the graph.

Figure S6

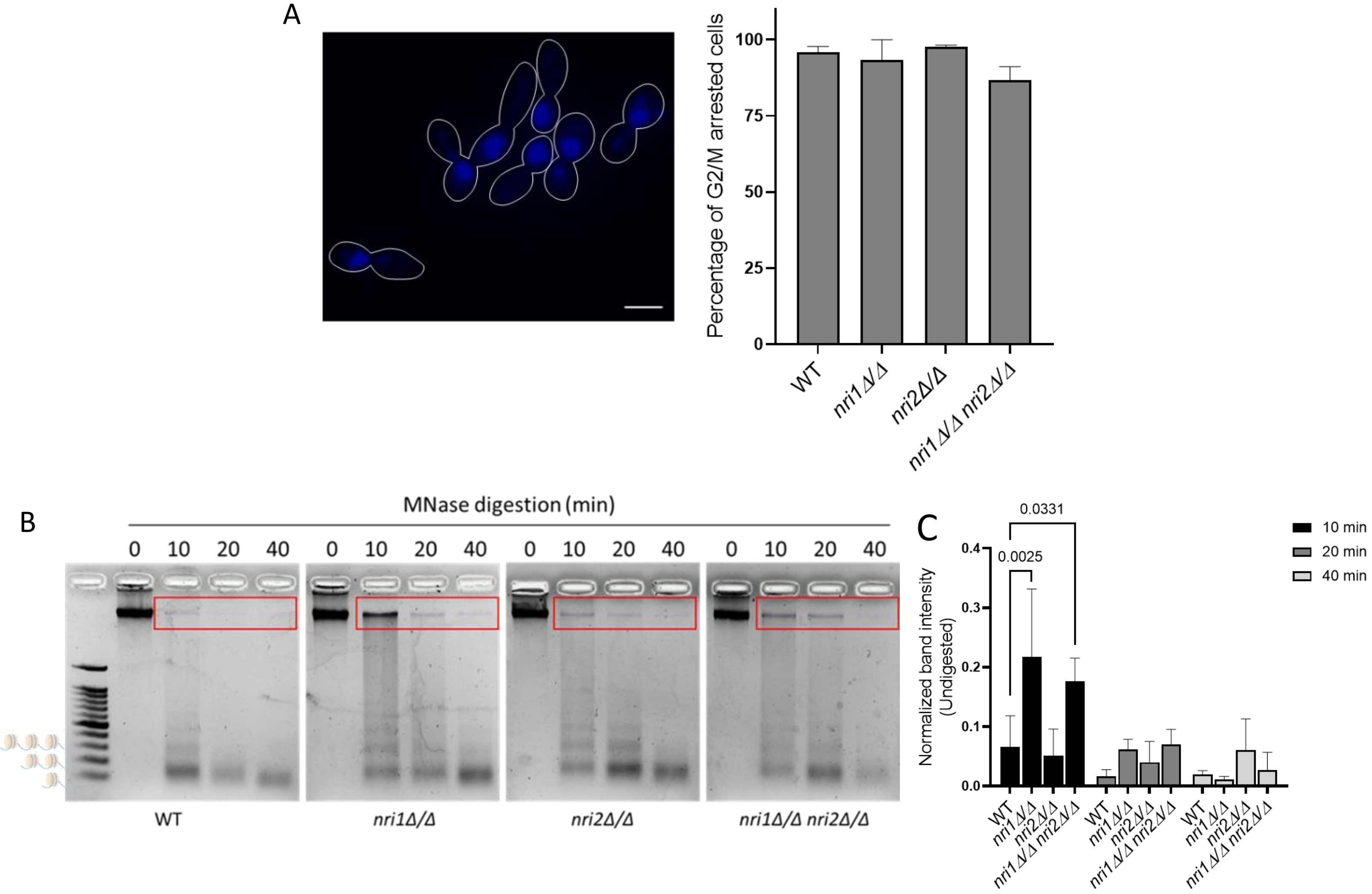

Figure S6: *nri1Δ/Δ* and *nri1Δ/Δ nri2Δ/Δ* mutants alter the chromatin architecture.
A. Left: representative images of the G2/M arrested cells having large bud with nucleus (DAPI) at the neck or stretched through the neck. Scale bar 2 µm. Right: percentage of G2/M arrested cells for the indicated strains after nocodazole treatment. Data are from three independent experiments. Error bars indicate standard deviation. Statistical analysis performed by one-way ANOVA. **B.** Nucleosome ladder patterns visualized on 2% agarose gel after MNase digestion of the chromatin for indicated duration from G2/M arrested cells of the indicated strains. First lane has 100 bp DNA ladder. **C.** Quantification of the intensities of the undigested bands (red box) for the indicated time points and strains. The intensities were normalized with corresponding 0 min band intensity for each strain. Data are from three independent experiments. Error bars indicate standard deviation. Statistical analysis performed by one-way ANOVA. p-value of < 0.05 was considered significant. Only significant p-values are mentioned in the graphs.

Figure S7

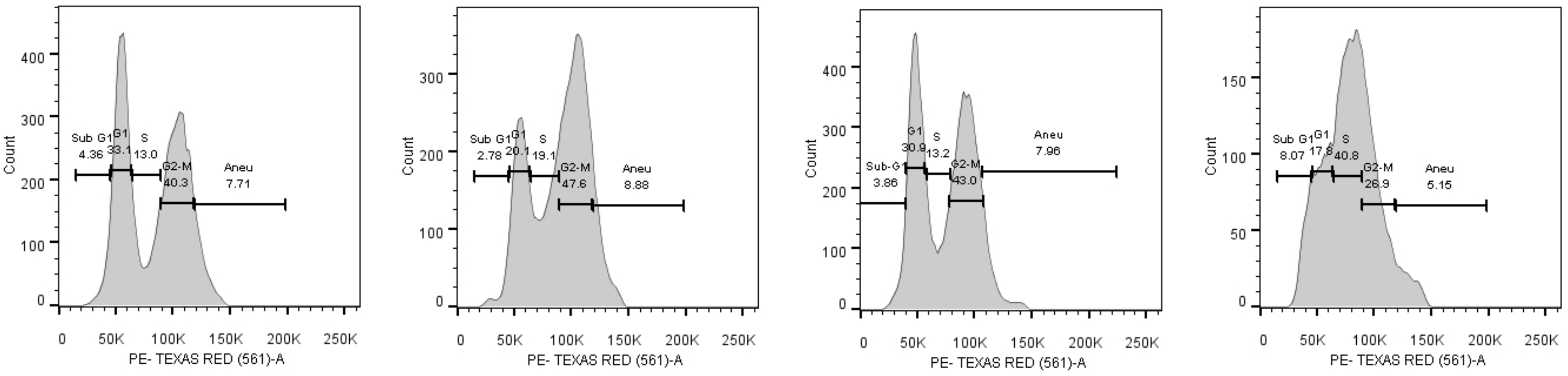

WT *nri1Δ/Δ* *nri2Δ/Δ* *nri1Δ/Δ nri2Δ/Δ*

Figure S7: Representative graphs showing gating parameters used to quantify FACS data.
To quantify the cell population at each cell cycle stage, gating was done according to Todd et al., 2018 by taking WT 2N and 4N peaks as reference (Left histogram) for *nri1Δ/Δ* and *nri1Δ/Δ nri2Δ/Δ* mutants. As *nri2Δ/Δ* showed left-ward shift in the peaks, gates were drawn according to 2N and 4N peaks. Gates were drawn at half-maximum height of 2N and 4N peaks corresponding to G1 and G2/M population (black horizontal I-beams), respectively. To quantify S phase population, a gate was drawn between the G1 and G2/M gates. Sub G1 population was quantified by drawing a gate before G1 gate and aneuploid population (Aneu) was quantified by drawing a gate after G2/M gate. The WT gate locations were then copied to mutant histograms to quantify cell cycle populations.

Figure S8

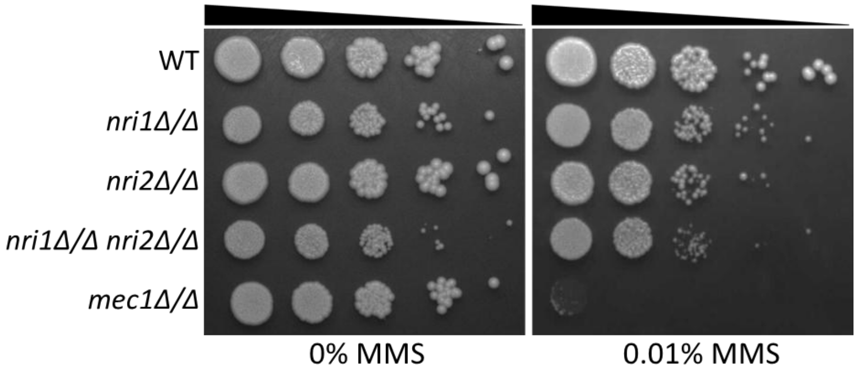

Figure S8: *nri* mutants does not alter susceptibility to MMS.
Image showing growth of 10-fold serially diluted cells spotted on the YPDU plate containing indicated concentration of MMS. Plates were incubated at 30 °C for 48 hrs and imaged.

Figure S9

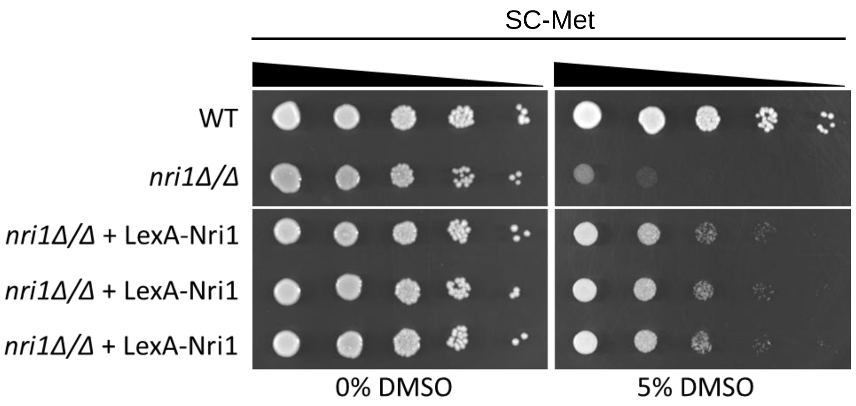

Figure S9: LexA-Nri1 fusion protein is able to rescue DMSO sensitivity in *nri1Δ/Δ* mutant.
Image showing growth of 10-fold serially diluted cells spotted on the SC-Met plate containing indicated concentration of DMSO. Cells from three independent transformants of *nri1Δ/Δ* + LexA-Nri1 were spotted, the plates were incubated at 30 °C for 48 hrs and imaged.

Figure S10

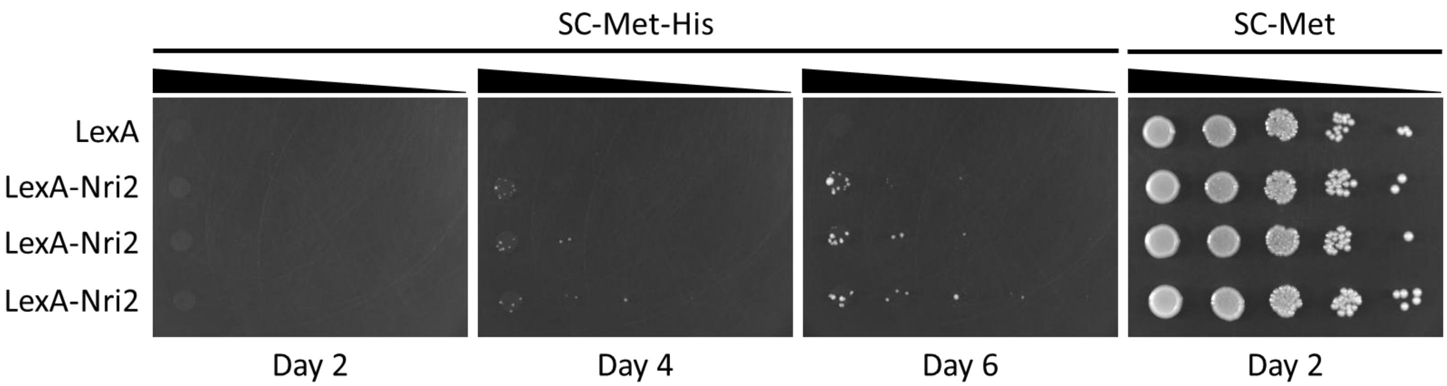

Figure S10: LexA-Nri2 fusion protein does not exhibit transcription activation potential. Image showing growth of 10-fold serial dilutions of the cells spotted on SC-Met-His and SC-Met plates. Plates were incubated at 30 °C for indicated days and plates were imaged.

Table S1: Strains used in this study

Sr. No.	Strain ID	Short genotype	Genotype	Strain background	Source
1	SN152	Wild type	*arg4Δ/arg4Δ leu2Δ/leu2Δ his1Δ/his1Δ URA3/ura3Δ::imm434 IRO1/iro1Δ::imm434*	SN148	Noble et al. 2010
2	SN250	Wild type	*arg4Δ/arg4Δ::CdARG4 leu2Δ::CdHIS1/leu2Δ::CmLEU2 his1Δ/his1Δ URA3/ura3Δ::imm434 IRO1/iro1Δ::imm434*	SN425	Noble et al. 2010
3	SGY12004	*nri1Δ/Δ*	*arg4Δ/arg4Δ leu2Δ/leu2Δ::ARG4 his1Δ/his1Δ URA3/ura3Δ::imm434 IRO1/iro1Δ::imm434 nri1Δ::HIS1/nri1Δ::LEU2*	SN152	Balachandra et al. 2020
4	SGY12006	*nri1Δ/Δ* + *NRI1*	*arg4Δ/arg4Δ leu2Δ/leu2Δ::NRI1-ARG4 his1Δ/his1Δ URA3/ura3Δ::imm434 IRO1/iro1Δ::imm434 nri1Δ::HIS1/nri1Δ::LEU2*	SGY12004	Balachandra et al. 2020
5	SGY12008	*nri2Δ/Δ*	*arg4Δ/arg4Δ leu2Δ/leu2Δ::ARG4 his1Δ/his1Δ URA3/ura3Δ::imm434 IRO1/iro1Δ::imm434 nri2Δ::HIS1/nri2Δ::LEU2*	SN152	Balachandra et al. 2020
6	SGY12010	*nri2Δ/Δ* + *NRI2*	*arg4Δ/arg4Δ leu2Δ/leu2Δ::NRI2-ARG4 his1Δ/his1Δ URA3/ura3Δ::imm434 IRO1/iro1Δ::imm434 nri2Δ::HIS1/nri2Δ::LEU2*	SGY12008	Balachandra et al. 2020
7	SGY12125	*nri1Δ/Δ nri2Δ/Δ*	*arg4Δ/arg4Δ leu2Δ/leu2Δ::ARG4 his1Δ/his1Δ URA3/ura3Δ::imm434 IRO1/iro1Δ::imm434 nri1Δ::FRT/nri1::FRT nri2Δ::HIS1/nri2Δ::LEU2*	SGY12008	This study
8	J110	*mad2Δ/Δ*	*ura3::imm434/ura3::imm 434 iro1/iro1::imm434 his1::hisG/his1::hisG leu2/leu2, mad2::LEU2/mad2::ARG4*	SN148	Thakur and Sanyal 2011
9	SGY12235	*mad2Δ/Δ nri1Δ/Δ*	*ura3::imm434/ura3::imm 434 iro1/iro1::imm434 his1::hisG/his1::hisG leu2/leu2, mad2::LEU2/mad2::ARG4 nri1Δ::FRT/nri1Δ::FRT*	J110	This study
10	SGY12366	*mad2Δ/Δ nri1Δ/Δ nri2Δ/NRI2*	*ura3Δ-iro1Δ::imm434/ ura3Δ-iro1Δ::imm434, his1Δ/his1Δ, arg4Δ/arg4Δ, leu2Δ/leu2Δ, mad2Δ::ARG4/mad2Δ::LEU2 nri1Δ::FRT/nri1Δ::FRT nri2Δ::FRT/NRI2*	SGY12235	This study
11	SGY12356	*nri1Δ/Δ nri2Δ/NRI2*	*arg4Δ/arg4Δ his1Δ/his1Δ URA3/ura3Δ::imm434 IRO1/iro1Δ::imm434 leu2Δ/leu2Δ::ARG4 nri1Δ::HIS1/nri1Δ::LEU2 nri2Δ::FRT-SAT1-FLP-FRT/NRI2*	SGY12004	This study

12	DKCa596	*mec1Δ/Δ*	*mec1Δ::CdHIS1/mec1Δ::CdARG4*	SN76	Legrand et al. 2011
13	YJB10695	Mtw1-GFP	*ura3Δ::λimm434/ura3Δ::λimm434 his1::hisG/his1::hisG arg4::hisG/arg4::hisG MTW1-GFP-URA3/MTW1*	BWP17	Burrack et al. 2011
14	SGY12228	Mtw1-GFP *nri1Δ/Δ*	*ura3Δ::λimm434/ura3Δ::λimm434 his1::hisG/his1::hisG arg4::hisG/arg4::hisG MTW1-GFP-URA3/MTW1 nri1Δ::FRT/nri1::FRT*	YJB10695	This study
15	SGY12273	Mtw1-GFP *nri1Δ/Δ nri2Δ/Δ*	*ura3Δ::λimm434/ura3Δ::λimm434 his1::hisG/his1::hisG arg4::hisG/arg4::hisG MTW1-GFP-URA3/MTW1 nri1Δ::FRT/nri1Δ::FRT nri2Δ::FRT/nri2Δ::FRT*	SGY12228	This study
16	YJB10742	Dad2-GFP	*ura3Δ::λimm434/ura3Δ::λimm434 his1::hisG/his1::hisG arg4::hisG/arg4::hisG DAD2-GFP-URA3/DAD2*	BWP17	Burrack et al. 2011
17	SGY12324	Dad2-GFP *nri1Δ/Δ*	*ura3Δ::λimm434/ura3Δ::λimm434 his1::hisG/his1::hisG arg4::hisG/arg4::hisG DAD2-GFP-URA3/DAD2 nri1Δ::FRT/nri1::FRT*	YJB10742	This study
18	SGY12334	Dad2-GFP *nri1Δ/Δ nri2Δ/Δ*	*ura3Δ::λimm434/ura3Δ::λimm434 his1::hisG/his1::hisG arg4::hisG/arg4::hisG DAD2-GFP-URA3/DAD2 nri1Δ::FRT/nri1::FRT nri2Δ::FRT/nri2Δ::FRT*	SGY12324	This study
19	YJB8675	Cse4-GFP	*ura3::imm434/ura3::imm 434 iro1/iro1::imm434 his1::hisG/his1::hisG, CSE4/CSE4:GFP:CSE4*	BWP17	Joglekar et al. 2008
20	SGY12090	Cse4-GFP *nri1Δ/Δ*	*CSE4/CSE4:GFP:CSE4 ura3Δ::λimm434/ura3Δimm434 his1::hisG/his1::hisG arg4::hisG/arg4::hisG nri1Δ::FRT/nri1::URA3-FLP*	YJB8675	This study
21	SGY12148	Cse4-GFP *nri1Δ/Δ nri2Δ/Δ*	*CSE4/CSE4:GFP:CSE4 ura3Δ::λimm434/ura3Δimm434 his1::hisG/his1::hisG arg4::hisG/arg4::hisG nri1Δ::URA3-FLP/nri1Δ::SAT1-FLP nri2Δ::FRT/nri2Δ::FRT*	SGY12092	This study
22	SGY12131	Cse4-GFP Tub1-RFP WT	*ura3Δ::λimm434/ura3Δimm434 his1::hisG/his1::hisG arg4::hisG/arg4::hisG CSE4/CSE4:GFP:CSE4 TUB1/TUB1-RFP::ARG4*	YJB8675	This study
23	SGY12136	Cse4-GFP Tub1-RFP *nri1Δ/Δ*	*ura3Δ::λimm434/ura3Δimm434 his1::hisG/his1::hisG arg4::hisG/arg4::hisG nri1Δ::FRT/nri1::URA3-FLP CSE4/CSE4:GFP:CSE4 TUB1/TUB1::RFP*	SGY12090	This study

24	SGY12152	Cse4-GFP Tub1-RFP *nri1Δ/Δ nri2Δ/Δ*	*CSE4/CSE4:GFP:CSE4 ura3Δ::λimm434/ura3Δimm434 his1::hisG/his1::hisG arg4::hisG/arg4::hisG nri1Δ::URA3-FLP/nri1Δ::SAT1-FLP nri2Δ::FRT/nri2Δ::FRT TUB1/TUB1::RFP*	SGY12148	This study
25	YJB13024	CEN7::TetO TetR-GFP	*ura3Δ::λimm434/ura3Δ::λimm434 his1::hisG/his1::hisG arg4::hisG/arg4::hisG ORF19.1963::TetR-GFP-Nat::ORF19.1961/ ORF19.1963,ORF19.1961 TetO-HIS1::CEN7*	YJB10038	Burrack et al. 2013
26	SGY12097	CEN7::TetO TetR-GFP *nri1Δ/Δ*	*ura3Δ::λimm434/ura3Δ::λimm434 his1::hisG/his1::hisG arg4::hisG/arg4::hisG ORF19.1963::TetR-GFP-Nat::ORF19.1961/ ORF19.1963,ORF19.1961 TetO-HIS1::CEN7 nri1Δ::FRT/nri1::URA3-FLP*	YJB13024	This study
27	SGY12099	CEN7::TetO TetR-GFP *nri2Δ/Δ*	*ura3Δ::λimm434/ura3Δ::λimm434 his1::hisG/his1::hisG arg4::hisG/arg4::hisG ORF19.1963::TetR-GFP-Nat::ORF19.1961/ ORF19.1963,ORF19.1961 TetO-HIS1::CEN7 nri2Δ::FRT/nri2::URA3-FLP*	YJB13024	This study
28	SGY12193	CEN7::TetO TetR-GFP *nri1Δ/Δ nri2Δ/Δ*	*ura3Δ::λimm434/ura3Δ::λimm434 his1::hisG/his1::hisG arg4::hisG/arg4::hisG ORF19.1963::TetR-GFP-Nat::ORF19.1961/ ORF19.1963,ORF19.1961 TetO-HIS1::CEN7 nri1Δ::URA3-FLP/nri1Δ::FRT nri2Δ::FRT/nri2Δ::FRT*	SGY12099	This study
29	SGY12275	CEN7::TetO TetR-GFP Tub1 WT	*ura3Δ::λimm434/ura3Δ::λimm434 his1::hisG/his1::hisG arg4::hisG/arg4::hisG ORF19.1963::TetR-GFP-Nat::ORF19.1961/ ORF19.1963,ORF19.1961 TetO-HIS1::CEN7 TUB1/TUB1-RFP::ARG4*	YJB13024	This study
30	SGY12285	CEN7::TetO TetR-GFP Tub1-RFP *nri1Δ/Δ*	*ura3Δ::λimm434/ura3Δ::λimm434 his1::hisG/his1::hisG arg4::hisG/arg4::hisG ORF19.1963::TetR-GFP-Nat::ORF19.1961/ ORF19.1963,ORF19.1961 TetO-HIS1::CEN7 nri1Δ::FRT/nri1::URA3-FLP TUB1/TUB1-RFP::ARG4*	SGY12275	This study
31	SGY12288	CEN7::TetO TetR-GFP Tub1-RFP *nri1Δ/Δ nri2Δ/Δ*	*ura3Δ::λimm434/ura3Δ::λimm434 his1::hisG/his1::hisG arg4::hisG/arg4::hisG ORF19.1963::TetR-GFP-Nat::ORF19.1961/ ORF19.1963,ORF19.1961 TetO-HIS1::CEN7 nri1Δ::URA3-FLP/nri1Δ::FRT nri2Δ::FRT/nri2Δ::FRT TUB1/TUB1-RFP::ARG4*	SGY12285	This study
32	SGY4034	*S. cerevisiae rad52Δ*	*MATa ura3-1 leu2,3-112 his3-1 trp1-1 ade2-1 can1-100 rad52Δ::KanMx*	W303	SKG lab

33	SC2H3	*5xLexO-HIS1*	*5xLexAOp-ADH1b/HIS1 5xLexAOp-ADH1b/lacZ*	SN152	Stynen et al. 2010
34	SGY12263	*EV LexA*	*5xLexAOp-ADH1b/HIS1 5xLexAOp-ADH1b/lacZ XOG1::pMET3-LexA-HA::HOL1*	SC2H3	This study
35	SGY12256	*LexA-NRI1*	*5xLexAOp-ADH1b/HIS1 5xLexAOp-ADH1b/lacZ XOG1::pMET3-LexA-HA-NRI1::HOL1*	SC2H3	This study
36	SGC2027	*nri1Δ/NRI1*	*arg4Δ/arg4Δ leu2Δ/leu2Δ his1Δ/his1Δ URA3/ura3Δ::imm434 IRO1/iro1Δ::imm434 nri1Δ::HIS1/NRI1*	SN152	Balachandra et al. 2020
37	SGY12269	*nri1Δ/Δ*	*arg4Δ/arg4Δ leu2Δ/leu2Δ his1Δ/his1Δ URA3/ura3Δ::imm434 IRO1/iro1Δ::imm434 nri1Δ::HIS1/nri1Δ::SAT1-FLP*	SN152	This study
38	SGY12308	*nri1Δ/Δ EV LexA*	*arg4Δ/arg4Δ leu2Δ/leu2Δ his1Δ/his1Δ URA3/ura3Δ::imm434 IRO1/iro1Δ::imm434 nri1Δ::HIS1/nri1Δ::SAT1-FLP XOG1::pMET3-LexA-HA::HOL1*	SGY12269	This study
39	SGY12348	*nri1Δ/Δ LexA-NRI1*	*arg4Δ/arg4Δ leu2Δ/leu2Δ his1Δ/his1Δ URA3/ura3Δ::imm434 IRO1/iro1Δ::imm434 nri1Δ::HIS1/nri1Δ::SAT1-FLP XOG1::pMET3-LexA-HA-NRI1::HOL1*	SGY12269	This study
40	SGY12382	*LexA-NRI2*	*5xLexAOp-ADH1b/HIS1 5xLexAOp-ADH1b/lacZ XOG1::pMET3-LexA-HA-NRI2::HOL1*	SC2H3	This study

Table S2: Plasmids used in this study

**Sr. No.**	**Strain ID**	**Plasmid description**	**Source**
1	pSFS2A	NAT flipper plasmid	Reuß et al. 2004
2	pSFS2A-NRI1-USDS	NAT flipper plasmid for *NRI1* deletion	This study
3	pSFS2A-NRI2-USDS	NAT flipper plasmid for *NRI2* deletion	This study
4	pSFU1	URA flipper plasmid	Morschhäuser et al. 1999
5	pSFU1-NRI1-USDS	URA flipper plasmid for *NRI1* deletion	This study
6	pBS-TUB1-RFP-ARG4	Tub1-RFP tagging plasmid	Varshney and Sanyal 2019
7	pSN-ARG4	ARG marker complementation plasmid	Noble et al. 2010
8	pC2HB	Bait plasmid	Stynen et al. 2010

9	pC2HB-NRI1	*NRI1* ORF cloned in pC2HB plasmid	This study
10	pC2HB-NRI2	*NRI2* ORF cloned in pC2HB plasmid	This study

Table S3: Primers used in this study

**Sr. No.**	**Primer name**	**Primer sequence**	**Description**
1	AJ1	GAGACGGGGTACCCCAATAAGCTAGTCTTAAATTATAGA	FP to amplify 572bp of *NRI1* US region for cloning
2	AJ2	GAGACCGCTCGAGTGTATGAATATGGGAGAAGG	RP to amplify 572bp of *NRI1* US region for cloning
3	AJ3	GAGAAAGGAAAAAAGCGGCCGCAAGTGTAAATGTAAGTCTAAGTGTATATTATT	FP to amplify 486bp of *NRI1* DS region for cloning
4	AJ4	GAGACGAGCTCTTGTAATTTGTTCTCTCGTTAGA	RP to amplify 486bp of *NRI1* DS region for cloning
5	AJ5	TGGACGTAGAATATCCTTGC	Diagnostic FP for *NRI1* deletion
6	AJ6	GAGACGGGGTACCGACAGTTTTATTAAATTGACTCGAT	FP to amplify 350bp of

			NRI2 US region for cloning
7	AJ7	GAGACCGCTCGAGTGTGACGAGTGTGTAGTGG	RP to amplify 350bp of *NRI2* US region for cloning
8	AJ8	GAGAAAGGAAAAAAGCGGCCGCAGCTCCATAGAAGTAAGCGTT	FP to amplify 378bp of *NRI2* DS region for cloning
9	AJ9	GAGACGAGCTCGTGGCACCATTGATTAAGTT	RP to amplify 378bp of *NRI2* DS region for cloning
10	AJ10	GGGGAAATTAAATCGGAAC	Diagnostic FP for *NRI2* deletion
11	AJ62	GAGACTAGCTAGCATGGATGTTCGTCGTTGTC	FP to clone *NRI1* in pC2HB
12	AJ63	GAAGGCCTTTAAAATTGAAATACTGGATCCAAA	RP to clone *NRI1* in pC2HB
13	AJ97	GAGACTAGCTAGCATGGTAAAAAACACTCTGTATAAG	FP to clone *NRI2* in pC2HB
14	AJ98	GAAGGCCTTACGTACTTGTAGGTTGAGAT	RP to clone *NRI2* in pC2HB

15	P22	CACGCTAGACAAATTCTTCC	Diagnostic reverse primer for *SAT1* Flipper integration
16	SA5	GCAACAGGAGAGGTTATGAT	Diagnostic reverse primer for *URA* Flipper integration

Table S4: Log_2fold change for the candidate DEGs (Figure 3A)

Cluster	Gene	*nri1Δ/Δ*	*nri1Δ/Δ nri2Δ/Δ*
Cell cycle progression	*CRZ1*	0	1.321629
	RGD1	0	-0.87521
	APC11	-1.58589	-1.28243
Kinetochore	*DAD4*	0	-1.28462
	NSL1	0	-0.86776
Replication	*CDC45*	-0.68348	-0.82519
	RNR3	0	1.339664
	ORC1	-0.73127	-0.78235
Transcription	*MED9*	0	-0.92406
	KNS1	0	2.620029
DNA damage response	*RAD10*	-3.63976	-3.33699
	RAD6	-2.30016	-2.52419
	RAD14	1.029709	1.262279
	MEC3	2.100949	2.503566
Oxidative stress response	*SOD4*	2.948039	3.376536
	GTT13	3.69233	4.661925
	GST1	0	1.32021
	GRX1	0	-1.24606
Epigenetic regulators	*ARP8*	-1.34049	-1.34334
	HIR1	-0.96601	-1.00601
	RPD31	0	-1.11131
	SWC4	1.114644	1.640314
	RTT102	1.876433	2.214057
TFs	*HAP3*	2.431169	2.505139

	RON1	-1.24287	-1.18134
	SSN3	-3.39718	-3.50995
	STB5	0	-1.23778
	TEA1	1.579368	1.578997
	ZCF22	3.314853	3.295852
	ZCF25	2.723982	2.999533
	ZCF26	1.050748	1.412876
	CTA26	0	3.237752
	IRF1	0	1.321629

Table S5: RNA-seq data read mapping

**Sample name**				**# Raw reads**	**# Trimmed reads**	**Sum of all 4 lanes of Raw reads**
S1	WT R1	WT	L001	9417406	9312803	37004149
			L002	9226402	9125875	
			L003	9251845	9140226	
			L004	9108496	9002767	
S2	*nri1Δ/Δ R1*	nri1	L001	11416319	11294588	44929358
			L002	11194525	11076942	
			L003	11239374	11108907	
			L004	11079140	10955402	
S3	*nri2Δ/Δ R1*	nri2	L001	9164491	9065895	36007935
			L002	8963974	8869365	
			L003	9010294	8905026	
			L004	8869176	8769895	
S4	*nri1Δ/Δ nri2Δ/Δ R1*	nri1_2	L001	12714288	12572015	50065881
			L002	12490655	12353316	
			L003	12521369	12369420	
			L004	12339569	12196370	
S5	WT R2	WT	L001	10601212	10481783	41622109
			L002	10363626	10249011	
			L003	10420931	10294022	
			L004	10236340	10115504	
S6	*nri1Δ/Δ R2*	nri1	L001	12057158	11921245	47429567
			L002	11814276	11683525	
			L003	11872367	11727854	
			L004	11685766	11548570	
S7	*nri2Δ/Δ R2*	nri2	L001	12000435	11858275	47286541
			L002	11791498	11655628	

			L003	11838591	11686437	
			L004	11656017	11512127	
S8	*nri1Δ/Δ nri2Δ/Δ R2*	nri1_2	L001	12764799	12607162	50268802
			L002	12537312	12386139	
			L003	12572175	12405213	
			L004	12394516	12235827	
S9	WT R3	WT	L001	13189787	13037246	51863444
			L002	12912143	12765941	
			L003	12976071	12813353	
			L004	12785443	12631280	
S10	*nri1Δ/Δ R3*	nri1	L001	11221436	11085981	44217286
			L002	11040190	10909184	
			L003	11044228	10900886	
			L004	10911432	10774624	
S11	*nri2Δ/Δ R3*	nri2	L001	9665549	9544629	37976184
			L002	9449920	9334686	
			L003	9508199	9379392	
			L004	9352516	9231228	
S12	*nri1Δ/Δ nri2Δ/Δ R3*	nri1_2	L001	13335141	13173273	52374792
			L002	13043022	12886947	
			L003	13109889	12937809	
			L004	12886740	12722978	

References:

Balachandra, Vinutha K., Jiyoti Verma, Madhu Shankar, et al. 2020. “The RSC (Remodels the Structure of Chromatin) Complex of Candida Albicans Shows Compositional Divergence with Distinct Roles in Regulating Pathogenic Traits.” *PLOS Genetics* 16 (11): e1009071. https://doi.org/10.1371/journal.pgen.1009071.

Burrack, L.S., S.E. Applen Clancey, J.M. Chacón, M.K. Gardner, and J. Berman. 2013. “Monopolin Recruits Condensin to Organize Centromere DNA and Repetitive DNA Sequences.” *Molecular Biology of the Cell* 24 (18): 2807–19.

Burrack, L.S., S.E. Applen, and J. Berman. 2011. “The Requirement for the Dam1 Complex Is Dependent upon the Number of Kinetochore Proteins and Microtubules.” *Current Biology* 21 (10): 889–96.

Joglekar, A.P., D. Bouck, K. Finley, et al. 2008. “Molecular Architecture of the Kinetochore-Microtubule Attachment Site Is Conserved between Point and Regional Centromeres.” *The Journal of Cell Biology* 181 (4): 587–94.

Legrand, M., C.L. Chan, P.A. Jauert, and D.T. Kirkpatrick. 2011. “The Contribution of the S-Phase Checkpoint Genes MEC1 and SGS1 to Genome Stability Maintenance in Candida Albicans.” *Fungal Genetics and Biology* 48 (8): 823–30.

Morschhäuser, J., S. Michel, and P. Staib. 1999. “Sequential Gene Disruption in Candida Albicans by FLP-mediated Site-specific Recombination.” *Molecular Microbiology* 32 (3): 547–56.

Noble, S.M., S. French, L.A. Kohn, V. Chen, and A.D. Johnson. 2010. “Systematic Screens of a Candida Albicans Homozygous Deletion Library Decouple Morphogenetic Switching and Pathogenicity.” *Nature Genetics* 42 (7): 590–98.

Reuß, O., Å. Vik, R. Kolter, and J. Morschhäuser. 2004. “The SAT1 Flipper, an Optimized Tool for Gene Disruption in Candida Albicans.” *Gene* 341: 119–27.

Stynen, B., P. Dijck, and H. Tournu. 2010. “A CUG Codon Adapted Two-Hybrid System for the Pathogenic Fungus Candida Albicans.” *Nucleic Acids Research* 38 (19): 184–184.

Thakur, J., and K. Sanyal. 2011. “The Essentiality of the Fungus-Specific Dam1 Complex Is Correlated with a One-Kinetochore-One-Microtubule Interaction Present throughout the Cell Cycle, Independent of the Nature of a Centromere.” *Eukaryotic Cell* 10 (10): 1295–305.

Varshney, N., and K. Sanyal. 2019. “Aurora Kinase Ipl1 Facilitates Bilobed Distribution of Clustered Kinetochores to Ensure Error-free Chromosome Segregation in Candida Albicans.” *Molecular Microbiology* 112 (2): 569–87.
